# Accounting for uncertainty in conflict mortality estimation: an application to the Gaza War in 2023-2024

**DOI:** 10.1186/s12963-025-00422-9

**Published:** 2025-10-13

**Authors:** Ana C. Gómez-Ugarte, Irena Chen, Enrique Acosta, Ugofilippo Basellini, Diego Alburez-Gutierrez

**Affiliations:** 1 Department of Digital and Computational Demography, Max Planck Institute for Demographic Research (MPIDR), Rostock, Germany; 2 Centre for Demographic Studies (CED), Barcelona, Spain; 3 Kinship Inequalities Research Group, Max Planck Institute for Demographic Research (MPIDR), Rostock, Germany

## Abstract

The ongoing Gaza War has resulted in significant loss of life and intensified an existing humanitarian crisis. Despite increasing demand for accurate data, mortality estimates remain challenging due to the inherent ‘statistical fog of war’. Accurate quantification is hindered by incomplete reporting and uncertain age-sex distributions of casualties. Official death tolls are likely influenced by damaged infrastructure, security disruptions, and political motivations, complicating detailed demographic verification. Our study introduces a novel methodological approach—a Bayesian model incorporating novel priors—to explicitly account for measurement errors in mortality estimation by addressing reporting completeness and uncertainty in demographic distributions. We use these methods to estimate sex- and age-specific mortality patterns and associated life expectancy (LE) and LE losses due to direct conflict deaths from the Gaza War. We find that LE in Gaza was 42.3 (39.4-45.0) in 2023 and 40.4 (37.5-43.0) in 2024, corresponding to LE losses of 34.4 (31.7-37.3) and 36.4 (33.8-39.3) years, respectively, compared to a counterfactual scenario with no conflict-related deaths. This corresponds to 78,318 (70,614-87,504) conflict deaths by the end of 2024, reflecting a 14-fold increase in all-cause mortality during the conflict’s first year. The age–sex pattern of Gaza’s conflict deaths aligns with UN-IGME profiles from past genocides. To contextualize these estimates, we compare them with LE losses observed in the Gaza Strip, the West Bank, and all of Palestine between 2012 and 2019. Our estimates align with previously published work, after adjusting the reporting priors to ignore underreporting. Our versatile and robust framework for mortality estimation under conditions of data scarcity can inform future conflict research.

**Supplementary Information:**

The online version contains supplementary material available at 10.1186/s12963-025-00422-9.

## Introduction

Scholars and humanitarians rely on different metrics to represent the severity of armed conflicts. Raw death tolls are widely used because they are easily communicated and understood [39, 68]. Despite their simplicity, death tolls alone offer an incomplete picture of war’s true mortality impact. Crucially, focusing on the total number of casualties overlooks the age and sex distribution of conflict-related deaths and fails to contextualize these losses within the broader demographic makeup of the affected population. The same death toll can be distributed very differently. For example, conflict mortality is generally highest among young men, but in cases of mass violence and genocide, the burden of mortality can be more evenly distributed among all sexes and age groups [44]. Age-specific mortality rates capture this heterogeneity by dividing death tolls by the population at risk for each age and sex group. However, practitioners and the public often find it useful to have a single metric that quantifies the mortality effect of war. Life expectancy at birth (LE) is a convenient indicator to summarize mortality, accounting for the age and sex distribution of both deaths and the population [64]. It represents the average age at death for a cohort of individuals exposed to the current age-specific mortality rates throughout their entire lives [46]. LE is a widely used metric to assess the impact of age-specific mortality shocks on populations [24].

Estimating LE in the context of armed conflict can be difficult, even though the measure is not, in itself, data-intensive. Period LE requires only data on age- and sex-specific death and population counts in a given year. However, even this basic demographic information can be difficult to obtain in war settings given political instability, security concerns, infrastructure damage, and limited institutional capability, among other things [76]. In protracted conflicts, the quality of reported data can vary unpredictably over time [67]. This complicates reliable analysis and results in uncertain and contested estimates [48, 73]. The relevant actors—including governments, armed groups, and civil society organizations—often have political motivations that raise questions about their ability or willingness to generate reliable conflict data [21].

Despite these challenges, a substantial body of literature focuses on estimating mortality in the context of armed conflicts. Death tolls can be assessed using either retrospective or prospective approaches. Retrospective analyses rely on surveys [13, 14, 48] or other demographic data to estimate both direct and indirect mortality [2, 23, 47]. In contrast, prospective studies collect and analyze data as a conflict unfolds. Commonly used sources such as the Uppsala Conflict Data Program (UCDP) dataset [76] and the Armed Conflict Location and Event Data (ACLED) [66] estimate death tolls by integrating multiple contemporaneous data sources. Frequently, multiple datasets containing verified deaths are combined using statistical methods to estimate conflict-related mortality [25, 65, 77]. However, estimates of war death tolls rarely provide detailed information on the age and sex distribution of casualties [44], precluding direct estimation of LE.

The often severe data limitations in the context of recent or ongoing conflicts raise an important ethical question: Is it justifiable to produce LE estimates when data quality is known to be poor? Should we prioritize providing potentially biased estimates during the emergence of crises, or is it better to refrain from making estimates about an ongoing crisis altogether? These questions are challenging because prospective estimates of conflict mortality are often used to monitor conflicts and motivate policy decisions around them [39, 83]. Governments, humanitarian organizations, and the media also use mortality estimates to convey the scale of the humanitarian catastrophes to the broader public [69]. In this paper, we argue that mortality measurement is important and necessary, but that urgency is no excuse for a lack of methodological rigor. In particular, we urge scholars in the demography of conflict to incorporate uncertainty directly into mortality estimates by using statistical tools. This is essential given our reliance on noisy data of unknown quality. We illustrate our novel approach using the ongoing Gaza War as a case study and discuss how it can be applied to other settings.

### The current conflict in Palestine

The case of Palestine exemplifies the challenges faced when studying mortality in war contexts. The Gaza War, triggered by the October 7th, 2023 Hamas-led attack in Israel, has resulted in widespread fatalities and intensified the region’s ongoing humanitarian crisis. Direct conflict mortality has been mostly concentrated among non-combatants in the Gaza Strip [22]. In addition, most of the population has been internally displaced and faces limited access to food, water, shelter, sanitation, and essential health services [37].

Official death tolls have been contested due to damage to infrastructure and hospital buildings, limited resources, and challenges in verifying the true scale of fatalities. This has hindered both the accurate reporting of death counts and the identification of the deceased [81, 87]. Some argue that official figures are underreported, as they do not account for indirect deaths [38] or people reported missing under the rubble [85]. Others have suggested that data from the Gaza Ministry of Health [17] may be inflated for political reasons [72]. However, recent studies have found no evidence of systematic over-reporting in GMoH data [22, 28, 36].

Several studies have estimated the mortality implications of the ongoing Gaza War. [35] estimated mortality due to traumatic injury in Gaza during the first nine months of the war by implementing a capture-recapture analysis of multiple hospital lists. As part of the analysis, the authors estimated that the underreporting rate of true mortality was 41%. [70] estimated the number of excess deaths in Palestinian children living in Gaza due to the conflict during 2023 and found that both the probability of dying in childhood and the probability of being orphaned in childhood increased significantly, ranging from a six- to nine-fold increase compared to 2022. [22] combined multiple lists of partially verified deaths with UNRWA^1^ refugee registries and estimated that LE in the Gaza Strip during the first twelve months of the conflict had decreased by more than 30 years for both males and females. Recent results from the Gaza Mortality Survey—a large-scale household survey—suggest that conflict-related deaths from October 7, 2023 to January 5, 2025 substantially exceeded official reports, with the GMoH’s reported death toll approximately 35% below the survey’s central estimate [74]. While these analyses attempted to address the likely under- and over-reporting of official death counts, they did not model the reporting error directly nor incorporate the uncertainty surrounding the age-sex distribution of conflict-related deaths.

### This study

This study aims to provide a flexible approach for estimating conflict-related mortality in Palestine given multiple sources of data uncertainty in conflict settings where only raw death tolls are available. While various sources of uncertainty might exist, our emphasis here is on measurement errors–the difference between the true value of a variable and the value obtained through measurement, or as in this case, through data collection. Specifically, we focus on two of the most common sources of measurement errors that affect conflict mortality estimates under conditions of data scarcity. The first is uncertainty around the degree of over- or under-reporting of the total death toll. This is relevant in cases where the total death toll is contested and the available numbers are likely inflated or undercounted. The second is due to incomplete or missing information on the sex and age of the reported casualties. Without these characteristics, there is an inherent uncertainty in the derived or assumed age-sex distribution of conflict-related casualties. This can arise if, for example, only a raw death toll is known or if deaths are grouped by very wide age groups (e.g., 0–65 and 65+). Unless otherwise stated, when referring to sources of uncertainty in this paper, we refer specifically to these two sources.

We introduce a Bayesian model that specifies prior distributions for both the age distributions and the reporting rate. By assigning prior distributions to the unknown parameters, we can model their uncertainty and allow for this uncertainty to propagate from the two separate sources. Our approach enables us to estimate age- and sex-specific mortality rates that account for both sources of uncertainty. This framework provides both point estimates and credible intervals for mortality rates, which can then be used to compute LE. In this regard, our work is similar to that of [71], who proposed a Bayesian model to estimate subnational mortality rates while directly modeling under-reporting of deaths due to inadequate vital registration; however, our model accounts for uncertainty in the age distributions differently by drawing them from a transformed Normal prior (see Section "Priors"). The estimation of the true mortality rates is also handled differently in the two frameworks: [71] implements a TOPALS model to estimate the mortality rates [12], whereas our model computes them directly from the reported deaths, the probability of correct reporting, and the exposures (see Section "Overall mortality rate").

In this paper, we exemplify our approach for the case of Palestine at the national and sub-national levels, but the overarching modeling framework we introduce is flexible. The analysis is also fully reproducible and, along with this article, we publish the data and code needed to replicate our results and to adapt the model to other settings in the open-access repository https://github.com/realirena/uncertainty_quantification. In doing so, we answer a call made in this journal to develop reliable metrics to understand the impact of conflict in the context of poor-quality data [69].

## Methods

In this section, we describe a Bayesian model for estimating sex- and age-specific mortality rates in conflict settings, where available information is uncertain in terms of both magnitude–death tolls and its degree of over- or under-reporting– and distribution–missing or incomplete information on the age and sex of the fatalities. Additionally, we present the specific data used for the case of Palestine.

### Model

#### Overall mortality rate

We distinguish between mortality directly attributable to the conflict and mortality indirectly caused or unrelated to the conflict in order to estimate the overall mortality rate. Let $R^{c}$ be the total number of reported direct conflict-related deaths in a given year, and $R^{c}_{sx}$ be its disaggregation by sex *s* and age *x*. Since only $R^{c}$ is typically known, we compute $R^{c}_{sx} = R^{c} *\pi _{sx}$, where $\pi _{sx}$ is the proportion of conflict-related deaths with sex *s* in age *x* according to pre-estimated age-sex distributions.

To estimate the true conflict-related mortality rate ($\mu ^{c}_{sx}$), we use the formula:

1 $$\begin{aligned} \mu ^{c}_{sx} = \dfrac{D^c_{sx}}{E_{sx}} = \dfrac{R^c_{sx}}{p E_{sx}}, \end{aligned}$$

where $E_{sx}$ are the exposures for sex *s* and age *x*, and $D^c_{sx}$ is the true number of conflict-related deaths. The latter is an unknown quantity that can be estimated from the reported conflict-related deaths as $D^c_{sx}={R^c_{sx}}/{p}$, where *p* denotes the reporting rate. If $p<1$ ($p>1$), this indicates that the registered number of conflict-related deaths is lower (higher) than the true number of deaths.

The overall mortality rate ($\mu _{sx}$) for each age-sex group is obtained as $\mu _{sx} = \mu ^{c}_{sx}+\mu ^{nc}_{sx}$, where $\mu ^{nc}_{sx} = {D^{nc}_{sx}}/{E_{sx}}$ denotes the non-conflict-related mortality rate.

#### Priors

We assign prior distributions to the unknown parameters to express our uncertainty about the parameters, in this case the reporting rate and the age-sex distribution of the reported casualties, and to incorporate information from previous studies into the model.

**Prior for the reporting rate** ($\textbf{p}$) The reporting rate can be drawn from a shifted and scaled Beta distribution as follows:

2 $$\begin{aligned} \dfrac{p - c}{r} \sim \textsf{Beta}(\alpha , \beta ) \end{aligned}$$

where $c, r, \alpha , \beta$ are specified in advance and determine the boundaries of the distribution, as well as the shape and scale. By using a prior distribution, we are able to quantify uncertainty surrounding the reported death toll. This is especially important given that in such a conflict setting, reported death tolls are likely to only partially reflect the true conflict mortality toll. Furthermore, we can investigate how different specifications of this reporting rate distribution affect the mortality estimates (see Section "Sensitivity: reporting rate prior check"). Finally, we also note that *p* can be set to vary by age-sex group, e.g. $p_{sx}$, or be a global parameter across all age-sex groups. In our application, we have information on reporting rates by (coarse) age-sex groups and therefore allow *p* to vary by these groups (see Section "Deriving priors from data and previous studies" for more details).

**Prior for the age-sex distribution** ($\pi _{sx}$) Let $\pi _{sx}$ be the proportion of people in sex $s = F, M$ and age group $x=1,\dots ,n$, and let $\varvec{\pi }$ be the vector containing the proportions for all age groups $\varvec{\pi }= (\pi _{F1},.., \pi _{Fn},\pi _{M1},.., \pi _{Mn})$ and $\sum _{s}\sum _{x} \pi _{sx} = 1$. In a Bayesian framework, we can set a prior on $\varvec{\pi }$, which allows us to incorporate the uncertainty from the age distribution estimates. We specify an overly-parameterized transformed Normal prior, as recommended by [19]:

3 $$\begin{aligned} \pi _{sx} = \dfrac{e^{\theta _{sx}}}{\sum _{s}\sum _{x} e^{\theta _{sx}}}, \theta _{sx} \sim \mathcal {N}(\phi _{sx}, \sigma _{sx}), \end{aligned}$$

where $\phi _{sx}, \sigma _{sx}$ can either be estimated from the data [18] or parameterized by additional hyperpriors (see [19, 45] for examples). In our case, since there is little information regarding population level values of $\phi _{sx}, \sigma _{sx}$, we choose to set these values to match what available information exists. In the case of the UN-IGME age distributions (see section "Age-sex distributions"), as an example, the 95% intervals are estimated along with the means of the age distributions, meaning that we can directly use these as inputs for $\phi _x, \sigma _x$ on the log scale ($E(\log (\theta ))$ and $sd(\log (\theta )))$, respectively.

### Joint Distribution

Let $D = (E_{sx}, R^c)$ denote the observed data and $\Theta = (\pi _{sx}, p, \mu _{sx})$ denote the model parameters. For completeness, we let $p(\Theta )$ denote the prior distribution of the parameters in $\Theta$ (in practice, we set these values rather than specifying these hyperprior distributions). We can then write the joint distribution of *D* and $\Theta$ as:

4 $$\begin{aligned} P(\Theta , D) \propto&\prod ^{S}_{s=1} \prod ^{X}_{x=1} \dfrac{R^c\pi _{sx}}{E_{sx}} \times \left( \frac{[(p+c)/r]^{\alpha -1}\{1-([(p +c)/r]\}^{\beta -1}}{\textsf{Beta}(\alpha , \beta )} \right) \nonumber \\&\times \prod ^{S}_{s=1} \prod ^{X}_{x=1} \Big \{\frac{1}{\sqrt{2\pi \sigma _{sx}}}{\exp }\left( -\frac{1}{2}\left\{ \dfrac{\theta _{sx}-\phi _{sx}}{\sigma _{sx}}\right\} ^{2}\right) p(\Theta ). \end{aligned}$$

**Fig. 1:**
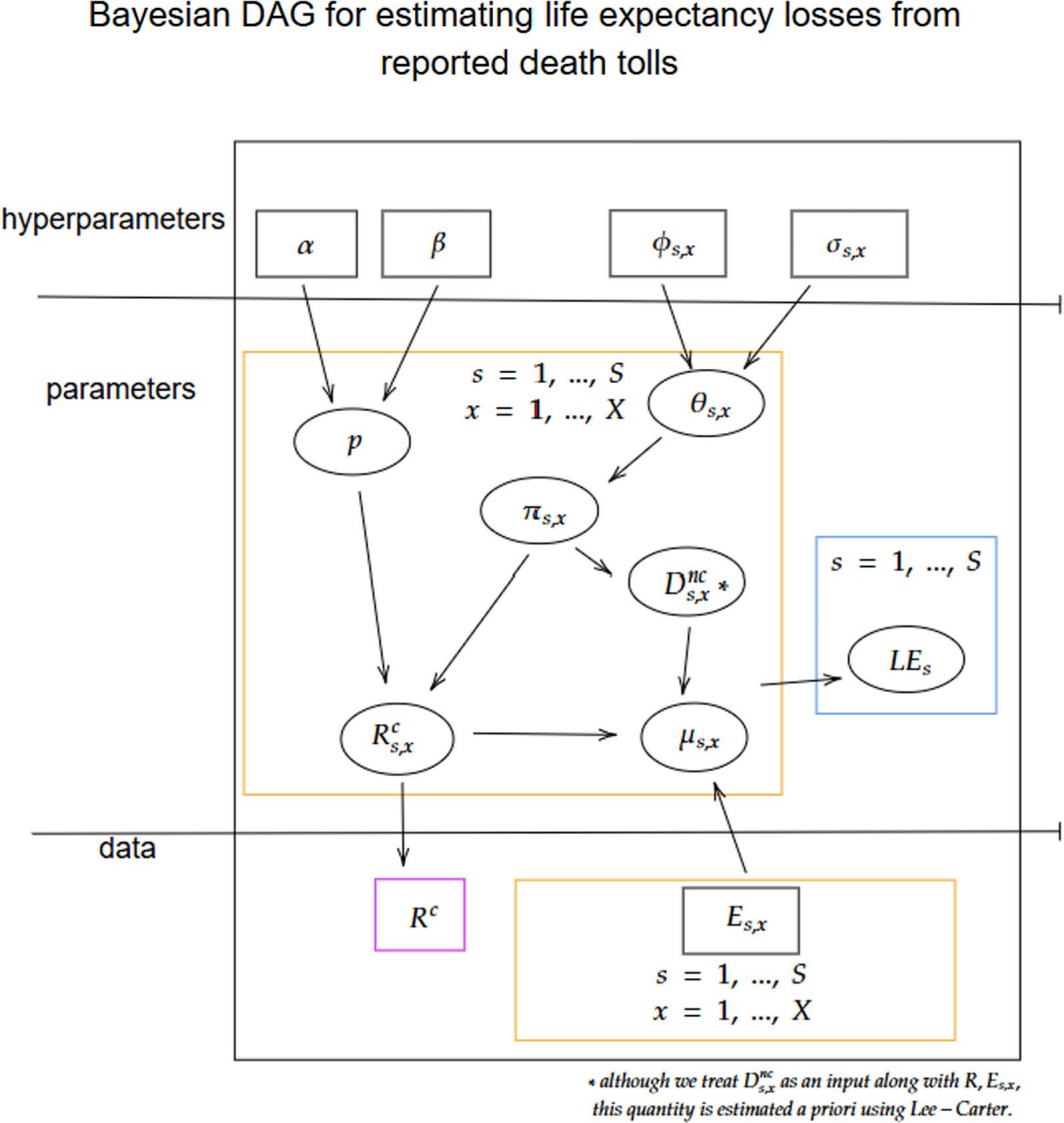
A directed acyclic graph of the Bayesian model used to estimate life expectancy (LE). The ovals represent unknown parameters and the rectangles represent the available data used as inputs. The colors of the boxes represent the different dimensions of the parameters and data, with purple denoting a scalar value, orange denoting multidimensional arrays (age-sex), and blue denoting a one dimensional array (sex). The hyperparameters for the reporting rate and the age distributions are specified based on information elicited from prior and current work. We note that $D_{s,x}^{c}$ before Oct. 7th, 2023 is not included in the DAG; however, it may impact the estimation of $\varvec{\pi }_{sx}$ (i.e. when estimating $\varvec{\pi }_{sx}$ from B’tselem’s historical average)

Figure 1 shows a directed acyclic graph (DAG) representation of our model and visualizes how the data and parameters relate to each other and produce the estimates of LE.

### Data

Table 1 provides an overview of the data sources we employ in our study, along with the type of data, and the age, sex, and regional breakdown availability for each source.

**Table 1:** Overview of data sources used in this study

**Data source**	**Type of information**	**Age & sex**	**Region specific**	**Temporal coverage**
PCBS^*a*^	Exposures	$\checkmark$	$\checkmark$	2012-2024
PCBS^*b*^	Vital registration death counts West Bank	$\checkmark$	$\checkmark$	2012-2017, 2023
UNPD^*c*^	Vital registration death counts Palestine	$\checkmark$		2012-2022
Guillot et al. (2025)^*d*^	Vital registration death counts and exposures Gaza	$\checkmark$	$\checkmark$	2017-2022
UN-IGME^*e*^	Infant and child death probabilities (1q0, 5q0)	$\checkmark$		2012-2022
B’Tselem^*f*^	Conflict deaths (individual-level records)	$\checkmark$	$\checkmark$	Gaza Strip: September 29^*th*^, 2000 - October 6^*th*^, 2023 West Bank: September 29^*th*^, 2000 - August 31^*st*^, 2024
OCHA^*g*^	Conflict deaths (aggregated tolls)	$\checkmark$	$\checkmark$	Gaza Strip: January 1^*st*^, 2012 - October 6^*th*^, 2023 West Bank: January 1 st, 2012 - December 31^*st*^, 2024
OCHA^*h*^	Conflict deaths (aggregated tolls) in the Gaza Strip		$\checkmark$	October 7^*th*^, 2023 - December 31^*st*^, 2024
GMoH^*i*^	Age-sex distributions of reported casualties	$\checkmark$	$\checkmark$	List 1: October 7^*th*^, 2023 - October 26^*th*^, 2023 List 7: October 7^*th*^, 2023 to October 7^*th*^, 2024
UN-IGME^*j*^	Age-sex patterns of crisis deaths	$\checkmark$		-

The temporal coverage refers to the information used for this study,not the time coverage of the data source. Sources: a. [49, 54, 57, 63] b. [55, 56, 63] c. [82] d. [22]; e. [80] f. [33]; g.[83, 85]; h. [84]; i. [1, 17]; j. [44]

#### Exposures ($E_{sx}$)

We use mid-year sex- and age-specific population estimates for the Gaza Strip and the West Bank as person-years of exposure to risk. These data were obtained from the Palestinian Central Bureau of Statistics (PCBS) for 2012–2024. To bridge differences between the 2007 and 2017 census-based estimates, we applied a General Additive Model (GAM) with P-splines to smooth the data. We note that obtaining age-sex exposure estimates can be challenging for many conflict settings, since the risk of displacement can substantially impact the population denominators.

#### All-cause death counts ($D_{sx}$) 2012-2022

Registered death counts by age and sex for Palestine (2012–2022) and the West Bank (2012–2017, 2023) were retrieved from the PCBS Statistical Yearbooks (2012–2024). Death counts for Gaza (2017–2022) were obtained from Guillot et al., [22]. The combination of these sources allowed us to construct a continuous series of sex- and age-specific death counts for 2012–2022 in Palestine, disaggregated by the Gaza Strip and the West Bank.

#### Conflict-related deaths before October 7th, 2023 ($D^c_{sx}$)

We retrieved from the Israeli Information Center for Human Rights in the Occupied Territories (B’Tselem) verified fatalities that occurred between September 29th, 2000, and October 6th, 2023, in Palestine by age, sex, and region. Independently, the United Nations Office for the Coordination of Humanitarian Affairs (OCHA) reports the aggregated number of validated conflict-related casualties since 2008. When compared to the number of casualties reported by OCHA, B’Tselem numbers are slightly lower. Therefore, we adjusted B’Tselem’s numbers, maintaining their age and sex distribution, to match those reported by OCHA. For 2023, all estimates include the conflict-related fatalities reported in B’Tselem that occurred between January 1st and October 6th, 2023.

#### Baseline mortality ($D^{nc}_{sx}$)

We used non-conflict mortality data to forecast the baseline mortality for 2023 and 2024, representing a counterfactual scenario in the absence of conflict. The forecast was generated using the Poisson version of the Lee-Carter method [7, 40]. We report results obtained by forecasting mortality using 2012-2019 as the fitting period (thereby excluding the effect of COVID-19) and data from PCBS; in the Supplementary Material, we perform a sensitivity analysis using a longer fitting period including the effects of COVID-19 (2012-2022).

#### Raw death toll after October 7th, 2023 ($R^c$)

For deaths that occurred after October 7th, 2023, we used the aggregate number of reported casualties reported by OCHA. From October 7th to December 31 st, 2023, there were 21,822 casualties recorded in the Gaza Strip [78, 83]. Additionally, there were 308 casualties in the West Bank [84]. OCHA additionally reports approximately 1,000 Hamas combatants killed in Israel on October 7th, 2023 [83]. For 2024, OCHA reported 23,719 casualties in the Gaza Strip [85], 498 in the West Bank [84] (see Table S1 in the Supplementary Material).

### Deriving priors from data and previous studies

As outlined in Section "Priors", using a Bayesian framework allows us to place priors on both the reporting rate and the age distributions, such that they reflect information from previous studies (e.g., [35, 44]).

#### Age-sex distributions

Since OCHA only reports total death counts for very wide age groups for the Gaza Strip, we derive priors for the age-sex casualties’ distributions from three separate sources: [17, 33], and the UN-IGME crisis mortality patterns [44]. Next, we describe the procedure to obtain the distribution from each data source.

**BTselem:** Annual age-sex distributions of conflict-related deaths from 2000 to 2022 are estimated from B’Tselem. Next, we estimate the mean and standard deviation of the proportion of casualties in each age-sex category. We excluded periods with relatively low conflict mortality. These estimates are used as parameters of normal distributions from where 1,000 random draws are taken to generate 95% confidence intervals of the proportion of casualties in each age-sex category.

**GMoH:** As of this writing (March 2025), the GMoH has publicly released seven cumulative lists of identifiable fatalities, including their identification numbers, names, ages, and sex. The first update included 6,746 identified casualties between October 7th, 2023, and Oct 26th, 2023, confirmed by hospitals. The 7th list, which contains 40,716 casualties, covers the period from October 7th, 2023, to October 7th, 2024, and combines hospital reports and deaths reported through an online survey launched by GMoH in an attempt to identify more casualties. Several studies have assessed the quality of the information contained in the lists and found no evidence of inflation or systematic errors in the information [22, 28, 35].

We obtain the proportion of deaths by age-sex distribution from the lists published by the GMoH. Confidence intervals were estimated by taking 1,000 random draws from a multinomial distribution using the proportions derived from each list. We use the 1st list for the 2023 estimates and the 7th list for the 2024 estimates, we retrieved the lists from [1].

**Fig. 2:**
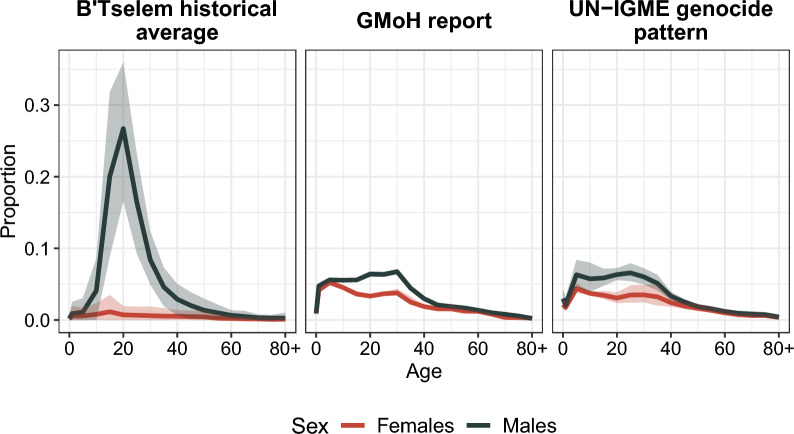
Age-sex distribution of conflict-related deaths for Palestine in 2023 based on the distributions: reported by the Gaza Ministry of Health (GMoH) [1, 17] on October 26th; ii) in 4 historical genocides (UN-IGME) [44]; and iii) reported by [33] between September 29th, 2000 and October 6th, 2023. *Source:* Authors’ elaborations on data from [17, 33, 44, 83]

**UN-IGME:** The UN-IGME crisis mortality patterns, which were derived from 164 crises in 57 countries, provide relative risks of mortality by age and sex for different types of crisis [44]. Here we use the genocide, conflict, and earthquake patterns. To account for the uncertainty from the UN-IGME patterns, for each age and sex, we took a random draw from a log-normal distribution with a mean equal to the log of the relative risks and standard deviation estimated from the given confidence intervals. We then exponentiated and multiplied each relative risk by its corresponding population exposure to obtain the number of conflict-related deaths, from which we obtained the proportion of conflict-related deaths by age and sex. We estimated 95% confidence intervals from 1,000 random draws. In the main text, we present results based on the UN-IGME genocide pattern, whereas the Supplementary Material presents alternative scenarios using the conflict and earthquake patterns. We chose the genocide pattern as it is the one that most closely resembles the mortality age-sex distribution in Gaza, as reported by [70] and seen in Fig. 2 (see also Figure S5 in the Supplementary Material).

Additionally, we use the UN-IGME combatant mortality pattern to distribute by age and sex the 1,000 Hamas combatants killed on October 7th, 2023 [83], who are included in the 2023 estimates for Gaza and Palestine.

Figure 2 shows the age-sex distributions for the scenarios presented here (see Supplementary Material for additional age-sex distributions). We see that in the historic pattern, conflict mortality is mostly concentrated among young males, reflecting a predominant combatant mortality during 2000-2022. According to this pattern, 92% of conflict-related deaths occur among males and 56% among males 20 to 40 years old. In contrast, the GMoH and UN-IGME genocide patterns are almost identical; in both patterns, conflict mortality risks are more evenly distributed across sex and age–40% of conflict mortality affects women–, although young males still experience a slight disadvantage–24% among males 20 to 40 years old.

#### Reporting rate

Our final choice of the upper and lower bounds of the reporting rate is informed by the estimated underreporting rates for certain age-sex intervals from [35]:

5 $$\begin{aligned} \dfrac{(p -LB_{s,x})}{UB_{s,x} - LB_{s,x}}\sim \textsf{Beta}(2,2), \end{aligned}$$

where $UB_{s,x}, LB_{s,x}$ correspond to the upper and lower 95% CIs associated with the underreporting rates for each available age-sex interval. In the absence of more detailed information, we chose to have each age-sex interval follow a (shifted and scaled) $\textsf{Beta}(2,2)$ distribution to have the prior be weakly informative at the mean (i.e., centering the probability density at the mean). In conflict settings where more detailed information about the particular shape of the reporting rate may be available, this can be easily modified to allow each age interval to have a different prior shape. In Section "Sensitivity: reporting rate prior check", we examine the sensitivity of the estimates to the different specifications of the reporting rate (see also Supplementary Material).

Figure S1 in the Supplementary Material shows the reporting rate prior for each age and sex group. We see that men have higher reporting rates than women, except in ages 15–29, which is expected as this is the age group with the highest male mortality. The lowest reporting rate is assumed to be among those 60 years and older, while the highest reporting rate is for males 45–59 years (86% mean reporting rate).

### Methodology for the West Bank

We have reasons to believe that the uncertainty in the reported conflict-related deaths for the West Bank is smaller, compared to Gaza. Both [33] and [84] have conducted exhaustive documentation and verification efforts to record casualties in the West Bank since October 7th, resulting in a high level of data reliability. Both organizations publish lists of verified casualties by age and sex. Given the minimal uncertainty associated with the age-sex distributions in the West Bank and the negligible reporting error for total West Bank deaths, our Bayesian approach was not required in this context. Instead, we employed a bootstrap method to estimate age- and sex-specific mortality rates. First, we obtain the aggregate number of casualties from [84] and the age-sex distribution from [33]. We then sample the reporting rate from a Uniform distribution with values between 0.8 and 1. The lower bound was chosen as a conservative threshold to account for potential underreporting of conflict deaths. As in Equation 1, we multiply the reporting rate by the aggregated casualties, the result is then distributed according to the age-sex distribution from [33]. Finally, we add the baseline deaths, which are sampled from a Poisson distribution, and divide by the exposures to obtain mortality rates. We repeat this procedure 1,000 times to obtain 95% CIs. National-level estimates are derived from a unified approach, estimated using the Bayesian model, with separate treatment of conflict-related deaths in Gaza and the West Bank.

## Results

In this section, we present the main results from our analysis using estimated age distributions from the three data sources. For each data source, we ran the Bayesian model with 4 chains for 2,000 iterations with 1,000 burnin. Convergence diagnostics were checked visually with traceplots and also by evaluating the estimated R-hat criterion [89]. All estimated R-hat values were $<1.01$, indicating that our model had achieved convergence. We implemented the model in Stan and R [9, 75] and provide all of the code and data used in the following https://github.com/realirena/uncertainty_quantification.

We computed life expectancies on 1,000 random posterior samples from each scenario and model run. We first present results at the national level (Palestine) and regional levels (Gaza Strip and West Bank). These results are estimated using GMoH age distributions for Palestine and Gaza with the prior of the reporting rate as defined in equation (5). For the West Bank, we use the age-sex distribution from B’Tselem and a Uniform distribution between 0.8 and 1 as the reporting rate prior. In Section "Gaza: comparison of data sources (GMoH, UN-IGME,Historical average)", we focus on the Gaza Strip, comparing estimates derived from three different age-distribution sources: GMoH reports, B’Tselem historical averages, and the UN-IGME genocide crisis. Additionally, we present estimated death toll and age-specific mortality rates in the Supplementary Material.

We estimated mortality for three periods: the calendar years 2023 and 2024, and the first full year of the war (October 7, 2023 to October 6, 2024). The 2023 and 2024 calendar-year estimates enable comparison with historical trends. However, the 2023 estimates combine nine months of low-level conflict with three months of intensive conflict, which may not accurately represent either period’s mortality experience. To address this limitation, we also estimated mortality for the first full year of the war (see Table 2).

### National and regional estimates

**Fig. 3:**
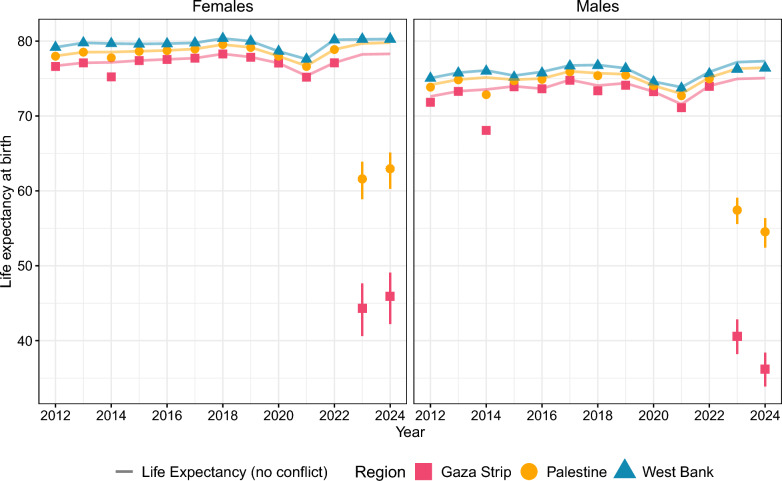
Life expectancy at birth at the national (in yellow) and regional levels using the GMoH age distributions for the Gaza Strip (in pink), and using B’Tselem’s age distribution the West Bank (in blue) for 2023 and 2024, with the prior of the reporting rate as defined in equation (5). The shapes indicate the observed life expectancy. The counterfactual scenarios of life expectancy without conflict deaths between 2012 and 2024 are indicated by the lines. For 2023 and 2024, the shapes refer to the mean value, and the intervals represent the 95% credible intervals

According to our model, the conflict-related death toll in Palestine was 38,172 (33,231–44,064) in 2023 and 41,617 (36,361–48,032) in 2024. This represents an $\sim$ 4 -fold increase in all-cause mortality (see Table S4 in the Supplementary Material). This implies a LE of 59.4 (57.2–61.3) years (61.6 (58.9–63.9) for females and 57.4 (55.6–59.1) for males), representing a decrease of 18.6 (16.6–20.8) years compared to the counterfactual scenario of no-conflict deaths (see Table 2). In 2024, LE for males decreased further to 54.5 (52.4–56.4), while for females it recovered slightly to 63.0 (60.3–65.1) (see Figure 3). This difference is largely due to the 7th GMoH list, which we are using to estimate 2024, having a higher proportion of male casualties compared to the 1 st GMoH list, used to produce the 2023 estimates.      

The vast majority of conflict-related deaths occurred in the Gaza Strip (∼8-fold increase in all-cause mortality); thus, when focusing on this region, we see even lower levels of LE. We estimated a LE of 40.6 (38.2–42.8) for males and 44.3 (40.6–47.6) for females in the Gaza Strip in 2023. This represents a LE loss of 34.4 (32.1–36.7) and 33.9 (30.6–37.6) years, respectively (see Table 2). Similar to the national level, in 2024, LE for females showed a slight increase compared to 2023, while it declined further for males. In contrast, despite experiencing also conflict death increases, LE in the West Bank remained relatively stable with no significant changes. Given the narrow range of the reporting probability assumed for the West Bank and the availability of a verified list of casualties from [33], the uncertainty associated with the West Bank estimates is lower than in Gaza.

**Table 2:** Life expectancy (LE) at birth and loss of life expectancy due to conflict in Palestine by sex and region using the sex-age distributions from GMoH for the Gaza Strip and B’Tselem for the West Bank, with the prior of the reporting rate as defined in equation (5). LE loss is estimated from a counterfactual based on Lee-Carter projections of mortality in the absence of the conflict. In column A, the values represent the mean of the annual LE estimates for 2012-2019. In columns B-D, the intervals represent the 95% credible intervals. Columns A-C estimate LE for the calendar year (Jan-Dec)

		A	B	C	D
Region	Sex	2012-2019	2023	2024	Oct.’23 - Sep.’24
**Life expectancy at birth (LE)**					
Gaza Strip	Females	77.2	44.3	45.9	34.3
			(40.6–47.6)	(42.2–49.1)	(30.9–37.5)
	Males	72.9	40.6	36.2	26.1
			(38.2–42.8)	(33.9–38.4)	(24.2–27.9)
	Total	75.0	42.3	40.4	29.3
			(39.4–45.0)	(37.5–43.0)	(26.8–31.7)
West Bank	Females	79.7	80.2	80.3	80.2
			(79.9–80.6)	(79.9–80.6)	(79.9–80.6)
	Males	75.9	76.3	76.4	75.9
			(75.9–76.6)	(76.1–76.7)	(75.5–76.2)
	Total	77.8	78.2	78.3	78.0
			(77.9–78.4)	(78.0–78.5)	(77.7–78.3)
Palestine	Females	78.7	61.6	63.0	53.6
			(58.9–63.9)	(60.3–65.1)	(50.3–56.7)
	Males	74.7	57.4	54.5	43.7
			(55.6–59.1)	(52.4–56.4)	(41.3–46.0)
	Total	76.7	59.4	58.4	48.0
			(57.2–61.3)	(56.1–60.4)	(45.2–50.7)
**Life expectancy loss**					
Gaza Strip	Females	0.3	33.9	32.4	43.9
			(30.6–37.6)	(29.2–36.0)	(40.7–47.4)
	Males	0.9	34.4	38.9	48.9
			(32.1–36.7)	(36.7–41.2)	(47.1–50.8)
	Total	0.6	34.4	36.4	47.4
			(31.7–37.3)	(33.8–39.3)	(45.0–49.9)
West Bank	Females	0.0	0.0	0.0	0.0
			(0.0–0.4)	(0.0–0.4)	(0.0–0.4)
	Males	0.1	0.9	0.9	1.4
			(0.6–1.3)	(0.6–1.3)	(1.0–1.8)
	Total	0.1	0.5	0.5	0.7
			(0.2–0.7)	(0.2–0.7)	(0.4–1.0)
Palestine	Females	0.1	18.1	16.8	26.1
			(15.8–20.8)	(14.7–19.5)	(23.1–29.4)
	Males	0.4	18.9	21.9	32.7
			(17.2–20.7)	(20.1–24.0)	(30.4–35.1)
	Total	0.3	18.6	19.7	30.0
			(16.6–20.8)	(17.7–22.0)	(27.4–32.8)

In the last column of Table 2 we include LE and LE loss estimates for the first full year of the war, that is, from October 7th, 2023 to October 6th, 2023. To calculate these, we average the exposures and the baseline mortality from 2023 and 2024. We estimate a LE of 26.1 (24.2–27.9) years for males and 34.3 (30.9–37.5) for females in Gaza during the first year of the war, which is around 10 year less than compared to the estimates for 2023 and 2024. This implies that the lifespan average for males was reduced to one third of the expected value in the absence of conflict, and more than half for females.

### Gaza: comparison of data sources (GMoH, UN-IGME, Historical average)

**Fig. 4:**
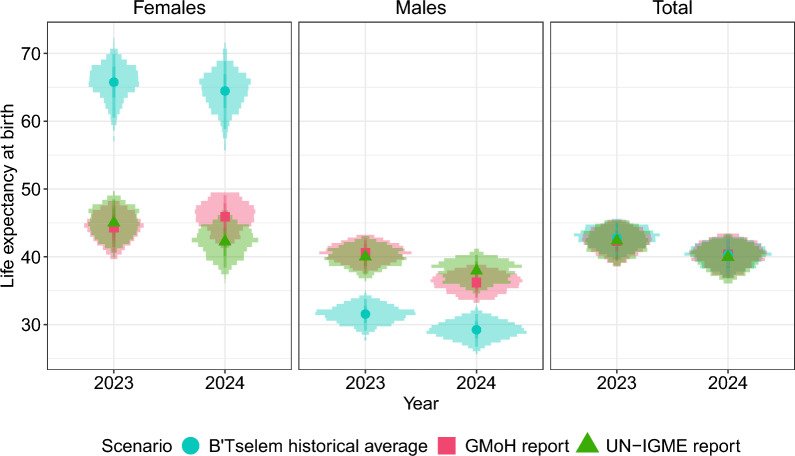
Life expectancy at birth for the Gaza Strip in 2023 and 2024 using the age distributions from 1) the GMoH (in pink) 2) B’Tselem historical average (in blue) 3) UN-IGME genocide crisis pattern (in green), with the prior of the reporting rate as defined in equation (5). The points refer to the mean value and the areas around the mean estimates represent the distribution of the posterior samples of life expectancy estimates. This graph corresponds to the zoomed-in area of Figure 3

To further understand the impact of the uncertainty around the age-sex distribution of deaths, we estimate LE in Gaza under the three different age distributions. Figure 4 displays the posterior samples of life expectancy estimates under three different scenarios in 2023 and 2024. Note that the plotted area corresponds to the zoomed-in panel in Figure 3, with an additional panel for the total population.

In Figure 4, we see large overlaps in the male LE estimates between GMoH reports and the UN-IGME genocide pattern. The scenarios based on the B’Tselem historical average age-sex distribution estimate higher LE for females and lower for males, consistent with the higher proportion of male deaths in the B’Tselem distribution (see Figure 2). Female LE estimates are more sensitive to the choice of age-sex distribution; the mean of the posterior distribution of LE in 2023 can range from 44.3 with the GMoH distribution to 65.8 with the historic distribution from B’Tselem. For 2024, the GMoH estimates of female LE increase, while for the other sources, it decreases. This is due to the lower proportion of female mortality in the 7th GMoH list of casualties. Interestingly, we see that the UN-IGME genocide estimates of LE for the total population (see right panel of Figure 4) essentially overlap those obtained when using the B’Tselem historical average and the GMoH reports. This is because the three distributions exhibit similar concentration of mortality in the middle age groups, despite differing proportions of deaths in each sex. Consequently, when the sex dimension is removed, the age patterns of all distributions converge, resulting in similar estimates.

As a sensitivity analysis, we used the age-sex UN-IGME patterns for conflicts and earthquakes (see Figures S5 and S6 in the Supplementary Material). Compared to Figure 4, we see a clear separation in the estimated 2023 LE for females between the GMoH age distribution and the UN-IGME conflict pattern. For males, among the UN-IGME patterns, the earthquake distribution has the greatest distance from the GMoH estimates.

### Sensitivity: reporting rate prior check

There are two sources of measurement uncertainty in our modeling framework: the uncertainty from the reporting rate of the death counts and the uncertainty from the age-sex distributions. To investigate how much of the uncertainty in the life expectancy estimates is due to the reporting rate, we conducted two sensitivity analysis.

For the first, we ran the model using the same reporting rate prior for all ages. This prior is based on the work of [37].Table S2 in the Supplementary Material displays the results after applying this prior. The results from this analysis are consistent with the main findings, although it yields narrower confidence intervals.

In the second sensitivity check, We first parameterized a reporting rate prior based on the “variant" scenarios in [22]. We selected a Beta distribution where the bounds of the distribution are set at 76% underreporting and 124% overreporting. These correspond to the “low" and “high" variants in [22] (see light blue distribution in Figure Figure S7 in the Supplementary Material). We then replicated our analysis (see Section "National and regional estimates") of the estimated LE in the Gaza Strip and Palestine for 2023 and 2024 using this prior. We do not change the reporting prior for the West Bank. Table S3 in the Supplementary Material shows the estimated LE by region after implementing the model with this new constructed prior.

LE estimates for Gaza with the updated prior are approximately 10 years higher than those shown in Table 2 for the total population in 2023 and 2024 (approximately 9 years for males and 12 years for females). This is expected as the new prior assumes less underreporting of deaths.

## Discussion

Monitoring mortality in conflict settings is essential, yet ensuring accurate and reliable estimates is both crucial and complex. Incorporating measurement uncertainty directly into mortality calculations is crucial given our reliance on noisy data of unknown quality. We developed a flexible approach for estimating life expectancy in crisis contexts, which combines uncertainty from two key sources: the degree of under- or over-reporting of the death toll and the age-sex distribution of deaths.

We implemented this approach to the case of Palestine. In 2023, LE was 59.4 years (42.3 in the Gaza Strip and 78.2 in the West Bank), representing a LE loss of 18.6 years (34.4 in the Gaza Strip and 0.5 in the West Bank) compared to a counterfactual scenario of no conflict-related mortality. Similar levels of LE and LE losses prevailed in 2024. As expected, our results reveal important differences in the impact of war in each region, which has important consequences for the future composition of each population.

This heterogeneity has important implications for public health. Conflict mortality tends to be highest in working-age men, but in cases of generalized violence, the burden of mortality is less selective and can be high among all age groups [44]. This matters for survivors, who may experience the loss of critical family and social support due to the death of members of their social network. Recent scholarship has highlighted that it is essential to understand the demographic composition of survivors bereaved by war in order to support orphans, widows, and bereaved parents [3, 6].

Our conflict-related death toll estimate of 78,318 (70,614-87,504) by the end of 2024 closely aligns with the independently conducted Gaza Mortality Survey’s estimate of 75,200 (63,600-86,800) violent deaths between October 7, 2023, and January 5, 2025 [74], providing robust mutual validation. Previous studies estimating LE for Palestine have assumed complete reporting of conflict-deaths [70] or have accounted for potential underreporting by estimating different scenarios (ie. assuming there are 10,000 individuals missing under the rubble who are unaccounted for [22]). To compare our method to these studies, we use a modified $\textsf{Beta}(3,3)$ prior (see Figure S7 in the Supplementary Material), for the reporting rate. When we apply this prior and limit our analysis to the period October 7th, 2023 to October 6th, 2023, the first year of the war, our results align with those of [22] with their central variant LE of 40.5 falling within our estimated interval of (36.6-42.4). We refer the reader to Supplementary Material Section 3.4 for further details. This indicates that 1) the uncertainty from the death counts is a key driver in estimating the final LE and loss of LE, and 2) our model is flexible enough to successfully reproduce previous estimates of LE by modifying the specification of the reporting error.

Our results indicate that, among the modeled sources of uncertainty, the specification of the prior on the underreporting rate contributes the most to uncertainty in the final LE estimates. This makes sense, given that the underreporting rate chosen for our analysis is allowed to range from 3% underreporting to 70% underreporting (meaning that the range of correctly reported deaths could be from just about one third of deaths to all deaths, depending on the age and sex group). The sensitivity analysis performed in Section "Sensitivity: reporting rate prior check" further investigates how changing the assumptions of the reporting rate prior can affect the life expectancy estimates.

In the case study analyzed here, uncertainty around the age-sex distribution of conflict-related deaths has minimal impact in total LE estimates, however it has great impact on the sex-specific LE estimates. For example, depending on the underlying age-sex distribution, the mean of the posterior LE for females in Gaza can change by almost 20 years depending on the choice of age-sex distribution in 2023.

There is far more data available on conflict-related deaths in the Hamas-Israel war than for other conflicts. Nonetheless, the overarching modeling framework presented here is flexible and can be adapted to other contexts (see section "Limitations and future work" for a more detailed discussion). The minimum data requirements are the total death toll and the population exposures, and the latter can be obtained from international databases (e.g., WPP). In contexts where the age-sex distribution of deaths is unknown, the age-sex patterns from the UN-IGME serve as a useful approximation. If the appropriate type of crisis is selected, the estimates can be very close to the true ones, as shown by our results when using the genocide pattern. When no additional information on the reporting rate is available, we recommend using a weak prior centered around one, to account for under- and over-reporting. This will likely increase the uncertainty around the estimates.

In the two first months of 2025, 2,864 conflict-related deaths were reported in the Gaza Strip [86, 87] in the West Bank [84]. Our estimates largely rely on population and death projections in the absence of conflict. Following the start of the conflict, this numbers could no longer be updated or verified. Consequently, the longer the conflict continues, the less reliable this data source becomes, introducing additional uncertainty. Although often overlooked, exposure estimates are equally important as the death estimates in the measurement of mortality. Therefore, future estimates for this conflict, and possibly for other contexts, would benefit from incorporating exposure uncertainty into the model.

### Limitations and future work

One limitation of our analysis is that our results depend on the estimation and forecast of non-conflict-related mortality, which we use as baseline to compute the excess mortality due to conflicts. With respect to estimation, our methodology does not currently include an assessment of its uncertainty; incorporating that uncertainty represents an important avenue for future work. With respect to forecasting, we employed the Poisson version of the Lee-Carter model on a rather short fitting period and independently for the national and sub-national setting. Although implausible outcomes are unlikely to emerge from 2-year forecasts (i.e. for 2023 and 2024), a coherent forecasting approach [e.g., 29, 42] could be employed to forecast mortality across multiple regions simultaneously.

We also note that the subnational estimates were analyzed separately, meaning that each scenario required a separate run of the model. Since the majority of the conflict deaths took place in the Gaza Strip, estimating these scenarios separately was sensible for our application. In the future, we could extend the Bayesian model to allow for joint national and subnational estimates via a hierarchical parameterization of the age distributions and possibly the reporting rates. A potential methodological extension to our our proposed reporting prior is to borrow the idea of a spike-and-slab shrinkage prior [32], where most of the probability mass is concentrated around a certain point (the “spike"), but values at the tail ends of the distribution are still possible (the “slab"). Specifying this prior would likely need more prior information or reasonable estimates about plausible reporting errors for the different age groups.

In a similar vein, we could also extend the model to estimate the true conflict mortality as a function of the multiple age distributions, rather than producing mortality estimates for each age distribution scenario. This approach could utilize existing methods for integration of multiple data sources (of varying quality) either by specifying the weighting scheme a priori [41, 92] or allowing the data to drive estimation of these weights [91]. In this framework, the main goal would be to arrive at the most likely estimate of the true conflict mortality given multiple data sources, which is a different research question than the one we sought to answer here. However, addressing this question would also be important for advancing methods for conflict mortality.

An additional limitation is that information on reporting rates is not available for all conflict settings. In our analysis, we use reporting rate estimates produced in a previous study [35], which applied capture–recapture analysis–also known as Multiple Systems Estimation (MSE)–to assess under-reporting based on the degree of overlap among multiple independent death lists [43]. This method enables researchers to quantify reporting error in the absence of complete data and can be used in various conflict mortality studies beyond Gaza. Existing applications include studies in Kosovo [5], Sudan [10], and Colombia [4]. In the Colombian case, the MSE-based estimates are also publicly accessible through an open-source R package [16], facilitating reproducibility and broader use.

For the West Bank, we sampled the reporting rate from a Uniform distribution bounded between 0.8 and 1.0. While the lower bound was chosen to represent a conservative threshold to account for potential underreporting, we acknowledge its inherent arbitrariness in the absence of empirical evidence. Nonetheless, given the relatively small proportion of conflict-related deaths occurring in the West Bank, this parameter choice does not substantially influence our findings.

A second, related approach is to draw on existing projects that compile and de-duplicate reports of violent deaths from multiple sources. For instance, the Uppsala Conflict Data Program [76] estimates conflict fatalities by combining NGO reports, international-organization data, historical archives, and other sources. Since 2013 the dataset has included (i) the number of distinct sources the coder consulted when deriving the best estimate and (ii) a low–high range for each violent event [30]. These metadata could help specify a more informative reporting prior by providing a death toll range and some idea of how credible (supported) the range is by the various sources. Exploring this possibility is promising, but further work is needed to assess whether the data can reliably support our model.

We also acknowledge that there could be conflict settings where historical information may not at all be appropriate to use in specifying a reasonable prior distribution for the reporting error. In this case, Bayesian estimation of conflict mortality might not be the most suitable approach, since the final estimates are influenced by the prior distributions, especially if the signal in the data is not strong enough to drive the estimation of posterior distribution. Although this is a limitation of the Bayesian approach, this also presents an opportunity to highlight the critical need for better understanding and estimation of conflict death reporting uncertainty, and to encourage ongoing research in this direction.

Finally, beyond the methodological uncertainty captured in our model, it is also important to acknowledge that our estimates do not capture the broader and potentially greater burden of indirect conflict mortality [35]. While our estimates of the impact of the first year of war on LE in Gaza and Palestine are substantial, they are likely to represent a lower bound of the true mortality burden. Our analysis focused exclusively on direct conflict-related deaths; however, the indirect effects of the war–often larger and more prolonged–remain unquantified in our estimates. Gaza’s population was already highly vulnerable before the war, with approximately 80% experiencing forced displacement and facing chronic shortages of medical supplies and healthcare [34]. Since October 2023, a convergence of factors–including indiscriminate destruction of civilian infrastructure, mass displacement, the collapse of public services, and severe restrictions on humanitarian access and funding [26, 90]–has triggered an unprecedented humanitarian crisis. Over 90% of the population has been forcibly displaced [79], and approximately 85% of hospitals, housing, and schools have been destroyed [11, 15, 88]. This destruction and the imposed restrictions have resulted in acute shortages of food, clean water [31], and essential medical services. The crisis has been further compounded by disease outbreaks [27], the erosion of public order–including reports of social unrest [8]. These conditions are expected to amplify excess mortality well beyond the toll of direct conflict deaths. The $\sim$8,000 excess non-violent death estimated by [74] from October 7, 2023 to January 5, 2025 provide critical short-term evidence on indirect mortality in Gaza. Medium-to-long-term consequences remain unquantified and could substantially elevate indirect mortality. [38] suggest that a conservative estimate would assume four indirect deaths for every direct death in Gaza. In other contexts marked by protracted conflict and systemic collapse, such as Sierra Leone’s civil war, this ratio has reached 16 to 1 [20]. The long-term demographic and health consequences of such indirect effects constitute a critical aspect of the war’s impact that lies beyond the scope of this study.

## Conclusions

Biased or incomplete data in conflict zones constrain our ability to produce accurate mortality estimates. Nevertheless, there is a pressing need for robust indicators–such as life expectancy estimates–that effectively assess and communicate the impact of conflicts. This tension between data limitations and the demand for informative metrics motivated this study. We demonstrate that these challenges need not be mutually exclusive; advances in statistical modeling allow us to partially account for the ‘statistical fog of war’ when estimating mortality rates. Our methodology shows that prospective mortality estimates should not shy away from discussing measurement uncertainty but instead incorporate it directly into the estimation procedure. We illustrate this approach with a case study of Palestine, where our results highlight the substantial impact of the Gaza War on the population, as evidenced by significant reductions in life expectancy at birth. Our analysis also revealed that Gaza’s estimated age–sex pattern of conflict deaths since October 7, 2023 mirrors UN-IGME profiles from prior genocides [44]. In an increasingly uncertain global landscape, timely estimates that account for data limitations will be ever more essential.

## Supplementary Information

Supplementary file 1

## Data Availability

The original data sets were obtained from the sources listed in Table 1. All data is publicly available and can be downloaded from each source. The codes needed to replicate our results and to adapt the model to other settings is available here: https://github.com/realirena/uncertainty_quantification.

## Footnotes

1 UN Relief and Works Agency for Palestine Refugees in the Near East.
