# Supplementary material for: Accounting for uncertainty in conflict mortality estimation: an application to the Gaza War in 2023-2024
Source: Popul Health Metr. 2025 Oct 13;23:55. doi: 10.1186/s12963-025-00422-9 (PMC12516881; doi:10.1186/s12963-025-00422-9)
Supplement: Supplementary file 1 [file 12963_2025_422_MOESM1_ESM.pdf]

# *Supplementary materials of:* Accounting for uncertainty in conflict mortality estimation: An application to the Gaza War in 2023-2024

Ana C. Gómez Ugarte[1], Irena Chen[1], Enrique Acosta[2,1], Ugofilippo Basellini[1], and Diego Alburez-Gutierrez[3]

[1] *Department of Digital and Computational Demography, Max Planck Institute for Demographic Research (MPIDR), Rostock, Germany.*
[2] *Centre for Demographic Studies (CED), Barcelona, Spain*
[3] *Kinship Inequalities Research Group, Max Planck Institute for Demographic Research (MPIDR), Rostock, Germany.*

September 15, 2025

## 1 Conflict-related death counts

Table S1 shows the conflict-related death counts used in the analysis.

| **Period** | **Region** | **Death counts** | **Source** |
|---|---|---|---|
| January 1st, 2023 - October 6th, 2023 | Gaza Strip | 34 | OCHA (2024a) |
| | West Bank | 198 | OCHA (2024a) |
| | Palestine | 232 | |
| October 7th, 2023 - December 31st, 2023 | Gaza Strip | 21,822 | Tech for Palestine (2025) |
| | West Bank | 308 | OCHA (2024a) |
| | Palestine | 22,130 | |
| January 1st, 2024 - December 31st, 2024 | Gaza Strip | 23,719 | OCHA (2024b) |
| | West Bank | 498 | OCHA (2024a) |
| | Palestine | 24,217 | OCHA (2023, 2024a) |
| October 7th, 2023 - October 6th, 2024 | Gaza Strip | 41,870 | Tech for Palestine (2025) |
| | West Bank | 719 | OCHA (2024a) |
| | Palestine | 42,589 | |

Table S1: Reported conflict-related death counts for different periods by region.

# 2 Model Specifications

## 2.1 Beta prior

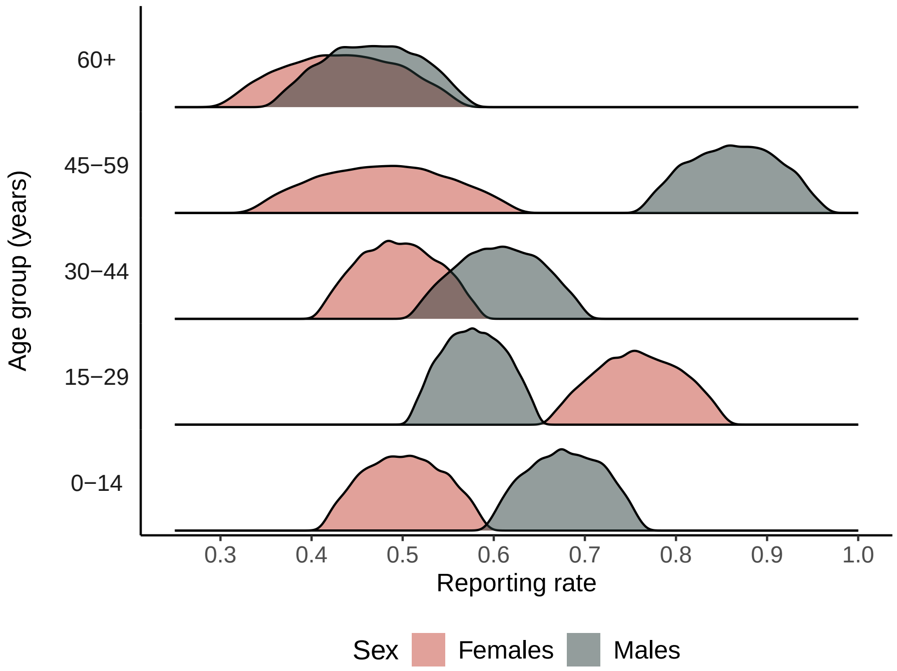


Figure S1: Visual representation of the Beta prior on the reporting rates derived from the age-sex specific underreporting rate estimated by Jamaluddine et al. (2025).

We chose to sample the reporting rate from a Beta distribution. A shifted and scaled Beta distribution allows us to easily represent the estimated reporting rate uncertainty as a prior. The second reason is that, when specified in a Bayesian context, having an unbounded reporting rate may result in model identifiability issues generated from the age distributions. Of course, other distributions can be specified for the reporting rate if desired; as an example, when we estimate the life expectancy for the West Bank (see Section 2.5 in the main text), we have reason to believe that the reported death tolls are much more accurate. Therefore, we use a Uniform distribution between 0.8 and 1 as the reporting rate prior for this scenario.

## 2.2 Prior age-sex distribution check

To verify the prior on the mortality distributions, we generated 10,000 random samples from the prior distribution in Equation 3 in the main text, where the means and standard deviations $(\phi_{xs}, \sigma_{xs})$ are derived from empirical information regarding the mortality distributions from the three sources (Gelman et al., 1996). We then compute the mean, standard deviation, and 95% quantiles of the 10,000 random samples and compare these quantities to the original values. Figures S2 displays these for the GMoH reported age distributions. In general, we can see the prior distribution recovers the mean mortality and the corresponding 95% quantiles for each age-sex group almost exactly.

For the historical age distributions from B'Tselem, there is much higher empirical uncertainty surrounding the age distributions (particularly for males). We found the model was able to recover most of the uncertainty after truncating the Normal distributions to the 95% empirical intervals, whereas when the distributions were not truncated, the model-generated uncertainty was much higher than the empirical uncertainty. We found similar results for the UN-IGME distributions (see Figure S4). We found that in both cases,

this did not significantly change the estimated life expectancies since the uncertainty from the reporting rate mainly drives the estimates, rather than the uncertainty from the age distributions. Because of this, we decided to use the results from the truncated model. Because the generated uncertainty from the GMoH age distributions was much smaller in magnitude compared to the other two sources (see Figure S5), we did not find it necessary to apply truncation to the prior. This truncation technique has been applied for this overparameterized prior distribution previously; see Gelman et al. (1996) for additional details.

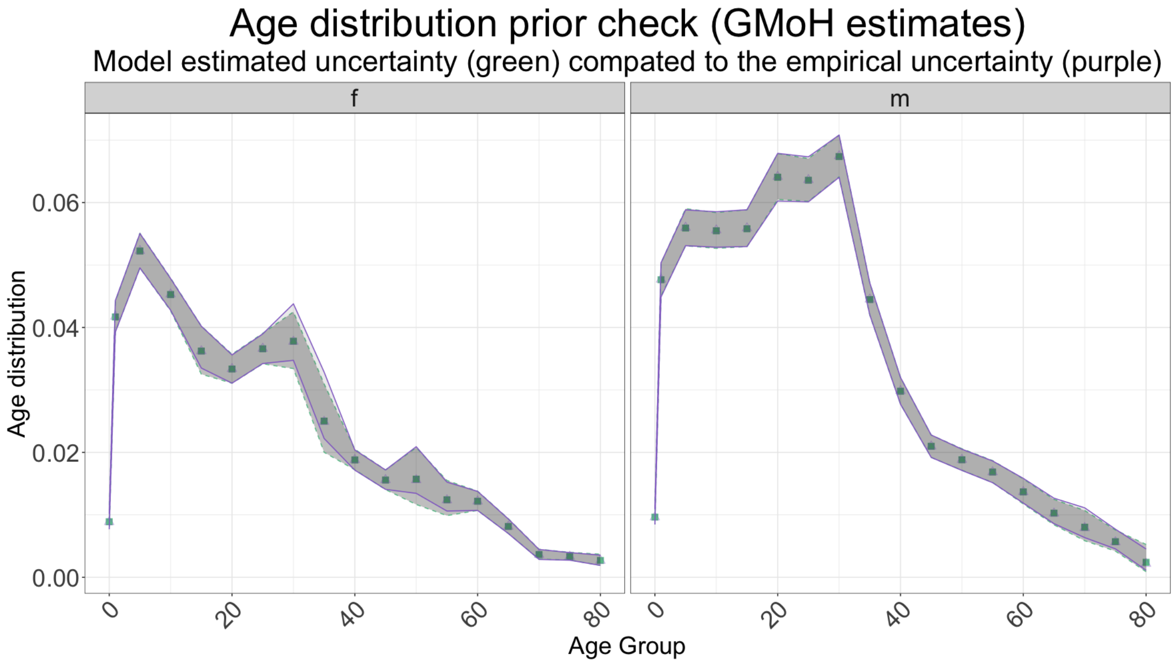


Figure S2: Comparison of model-generated uncertainty (green dashes) to empirical uncertainty (purple line) for the GMoH reported age distribution for 2023. From the figure, we can clearly see that the model-generated age distribution uncertainty matches the empirical uncertainty fairly well across all age groups.

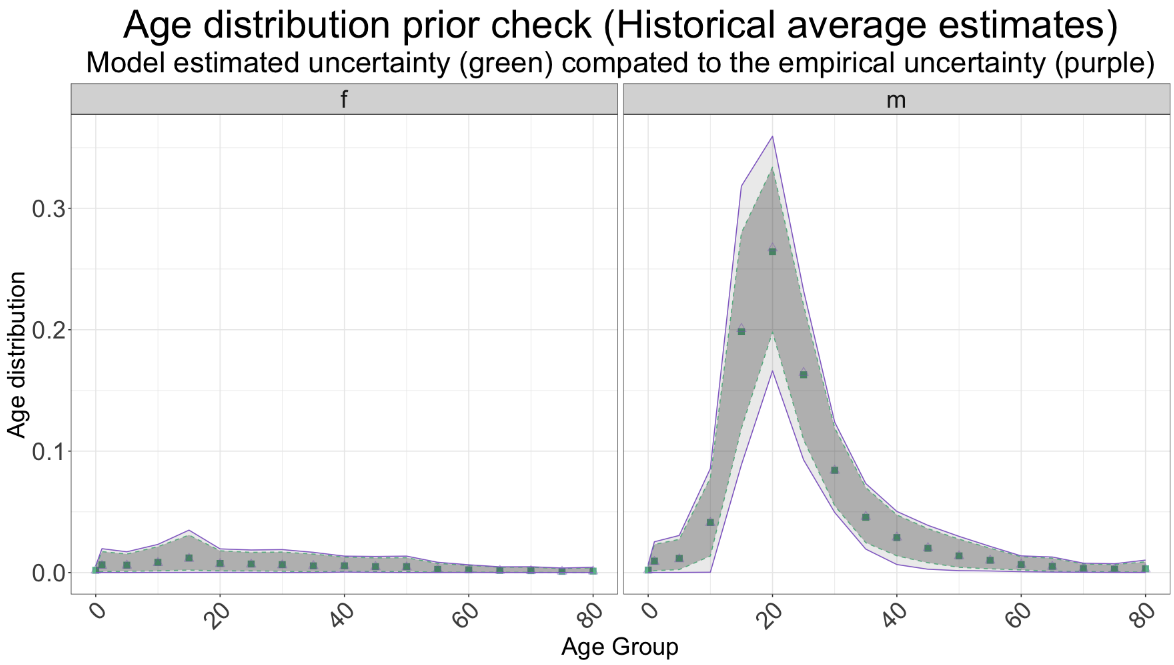


Figure S3: Comparison of model-generated uncertainty (green dashes) to empirical uncertainty (purple line) for the reported age distribution based on historical estimates from B'Tselem for 2023. From the figure, we can clearly see that the model-generated age distribution uncertainty recovers the means of the empirical age distribution. However, in general, the empirical uncertainty is slightly undercovered across all age groups.

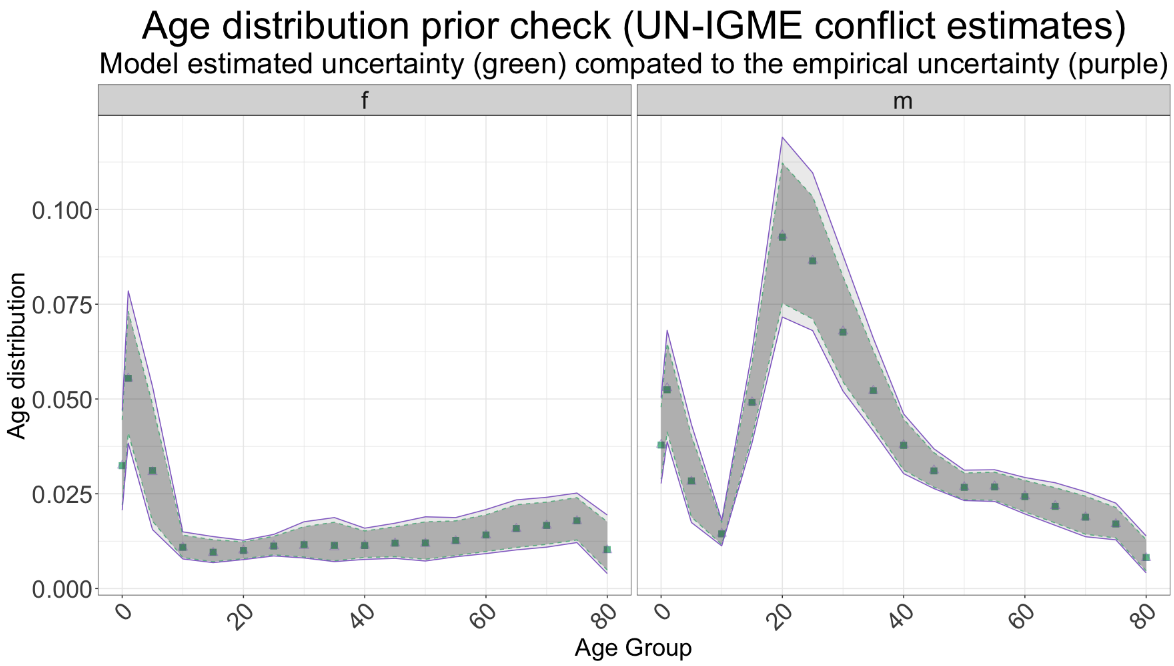


Figure S4: Comparison of model-generated uncertainty (green dashes) to empirical uncertainty (purple line) for the UN-IGME conflict pattern reported age distribution for 2023, along with the estimated means. From the figure, we can see that the empirical means of the age distributions (purple triangles) are recovered by the model (green squares), but that the model tends to underestimate the uncertainty (most likely due to truncating the prior distribution).

### 2.2.1 Additional age-sex distributions

Figure S5 shows the age-sex distributions for six scenarios: 1) B'Tselem historical average, 2) GMoH report from List 1, 3) GMoH report from List 7, 4) UN-IGME conflict pattern, 5) UN-IGME earthquake pattern, and 6) UN-IGME genocide pattern.

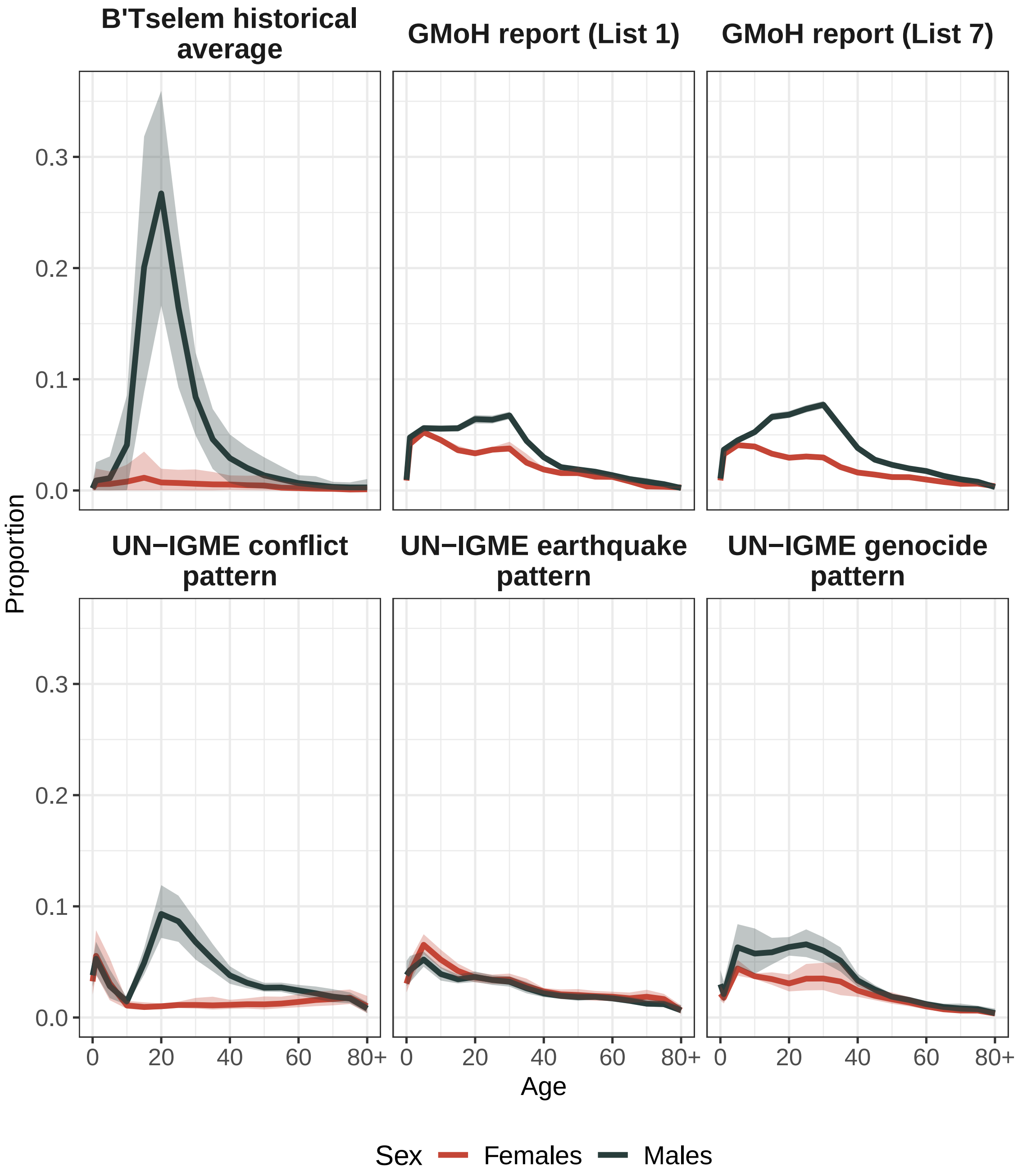


Figure S5: Age-sex distribution of conflict-related deaths for Palestine in 2023 based on the distributions: reported by the Gaza Ministry of Health (GMoH) (Airwars, 2024; GMoH, 2023); ii) in historical crisis (UN-IGME) (Mathers et al., 2023); and iii) reported by B'Tselem (2023) between September 29$^{th}$, 2000 and October 6$^{th}$, 2023.
*Source:* Authors' elaborations on data from GMoH (2023); B'Tselem (2023); Mathers et al. (2023); OCHA (2023).

# 3 Additional Analyses

In this section, we present the results for additional analysis and sensitivity checks of the results presented in the main text.

## 3.1 UN-IGME additional analyses

Figure S6 shows the LE and LE loss estimates for the Gaza Strip for five different age-sex distribution priors: 1) the GMoH 2) B'Tselem historical average 3) UN-IGME genocide crisis pattern 4) UN-IGME conflict crisis pattern and 5) UN-IGME earthquake crisis pattern.

Estimated total life expectancy loss tends to converge around the same value (roughly 39.8 years in 2023 and 40.2 years in 2024) under all distribution patterns. However, we see clear differences when disaggregating estimated LE and loss of LE by sex. The historic distribution gives the highest LE estimates for females and the lowest for males, due to mortality being highly concentrated among males. On the contrary, the highest male LE, and the lowest female LE, estimates come from the UN-IGME earthquake pattern, which assumes higher female mortality.

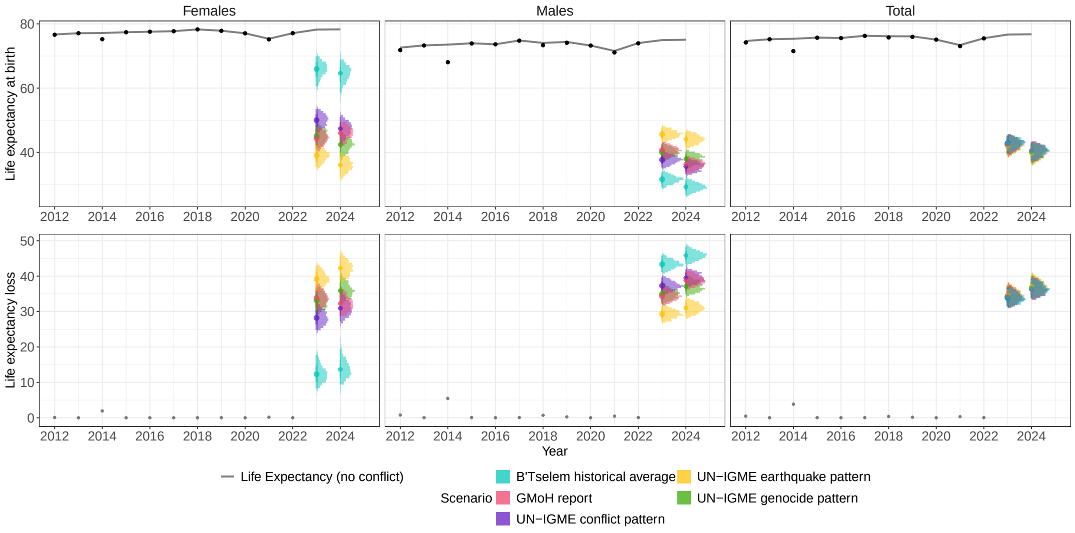


Figure S6: Life expectancy at birth and loss of life expectancy estimates for Gaza using the age distributions from 1) the GMoH 2) B'Tselem historical average 3) UN-IGME genocide crisis pattern 4) UN-IGME conflict crisis pattern and 5) UN-IGME earthquake crisis pattern.

## 3.2 Sensitivity analysis: constant reporting rate over age and sex

In this section, we present the results of the sensitivity analysis using the same reporting rate prior for all ages. This prior is based on the work of (Jamaluddine et al., 2024). Following Equation (5) in the main text, we allow the upper bound of the range to correspond to 13% underreporting, which was estimated to be the GMoH reporting accuracy before the conflict (see dark blue distribution in Figure S7). The results from this analysis are consistent with the main findings, although it yields narrower confidence intervals.

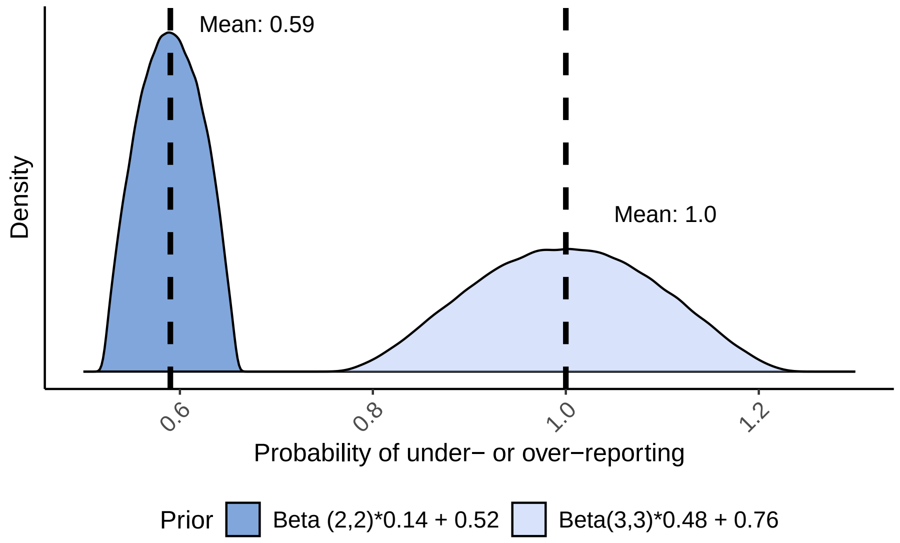


Figure S7: Visual representation of the Beta prior on the reporting rate. The dashed line represents the mean of each distribution. The dark blue distribution (left) corresponds to the underreporting rate estimated by Jamaluddine et al. (2025). The light blue curve (right) corresponds to a distribution parametrized based on the "variant" scenarios in Guillot et al. (2025).

| Region | Sex | A 2012-2019 | B 2023 | C 2024 | D Oct.'23 - Sep.'24 |
|---|---|---|---|---|---|
| **Life expectancy at birth (LE)** | | | | | |
| Gaza Strip | Females | 77.2 | 46.1 | 47.7 | 36.0 |
| | | [75.2-78.3] | (43.9-48.0) | (45.8-49.5) | (33.8-38.2) |
| | Males | 72.9 | 39.2 | 35.1 | 25.1 |
| | | [68.1-74.8] | (37.2-41) | (33.2-36.9) | (23.5-26.7) |
| | Total | 75.0 | 42.3 | 40.3 | 29.2 |
| | | [71.5-76.3] | (40.2-44.2) | (38.2-42.2) | (27.3-31.0) |
| West Bank | Females | 79.7 | 80.2 | 80.3 | 80.2 |
| | | [79.2-80.4] | (79.9-80.6) | (79.9-80.6) | (79.9-80.6) |
| | Males | 75.9 | 76.3 | 76.4 | 75.9 |
| | | [75.0-76.7] | (75.9-76.6) | (76.1-76.7) | (75.5-76.2) |
| | Total | 77.8 | 78.2 | 78.3 | 78 |
| | | [77.1-78.5] | (77.9-78.4) | (78.0-78.5) | (77.7-78.3) |
| Palestine | Females | 78.7 | 63.0 | 64.4 | 55.5 |
| | | [77.8-79.5] | (61.5-64.2) | (63.0-65.6) | (53.6-57.2) |
| | Males | 74.7 | 56.5 | 53.7 | 42.7 |
| | | [72.8-75.9] | (54.9-57.9) | (52.0-55.3) | (40.7-44.6) |
| | Total | 76.7 | 59.5 | 58.5 | 48.2 |
| | | [75.3-77.4] | (58.0-60.9) | (57.0-60.0) | (46.2-50.0) |
| **Life expectancy loss** | | | | | |
| Gaza Strip | Females | 0.3 | 32.2 | 30.6 | 42.2 |
| | | [0.0-1.9] | (30.2-34.4) | (28.8-32.5) | (40.1-44.5) |
| | Males | 0.9 | 35.8 | 40 | 49.9 |
| | | [0.0-5.5] | (33.9-37.8) | (38.1-41.9) | (48.3-51.5) |
| | Total | 0.6 | 34.4 | 36.5 | 47.6 |
| | | [0.0-3.8] | (32.5-36.5) | (34.6-38.5) | (45.7-49.4) |
| West Bank | Females | 0.0 | 0.0 | 0.0 | 0.0 |
| | | [0.0-0.0] | (0.0-0.4) | (0.0-0.4) | (0.0-0.4) |
| | Males | 0.1 | 0.9 | 0.9 | 1.4 |
| | | [0.0-0.2] | (0.6-1.3) | (0.6-1.3) | (1.0-1.8) |
| | Total | 0.1 | 0.5 | 0.5 | 0.7 |
| | | [0.0-0.1] | (0.2-0.7) | (0.2-0.7) | (0.4-1) |
| Palestine | Females | 0.1 | 16.7 | 15.4 | 24.2 |
| | | [0.0-0.8] | (15.4-18.2) | (14.2-16.7) | (22.6-26.1) |
| | Males | 0.4 | 19.8 | 22.7 | 33.6 |
| | | [0.0-2.3] | (18.4-21.4) | (21.1-24.4) | (31.8-35.7) |
| | Total | 0.3 | 18.4 | 19.6 | 29.8 |
| | | [0.0-1.6] | (17.1-20.0) | (18.1-21.1) | (28.0-31.8) |

Table S2: Life expectancy (LE) at birth and loss of life expectancy due to conflict in Palestine by sex and region using the sex-age distributions from GMoH for the Gaza Strip and B'Tselem for the West Bank, with the prior of the reporting rate shown in the dark blue distribution in Figure S7. LE loss is estimated from a counterfactual based on Lee-Carter projections of mortality in the absence of the conflict. In column A, the intervals refer to the minimum and maximum LE in the interval. In columns B-D, the intervals represent the 95% credible intervals. Columns A-C estimate LE for the calendar year (Jan-Dec).

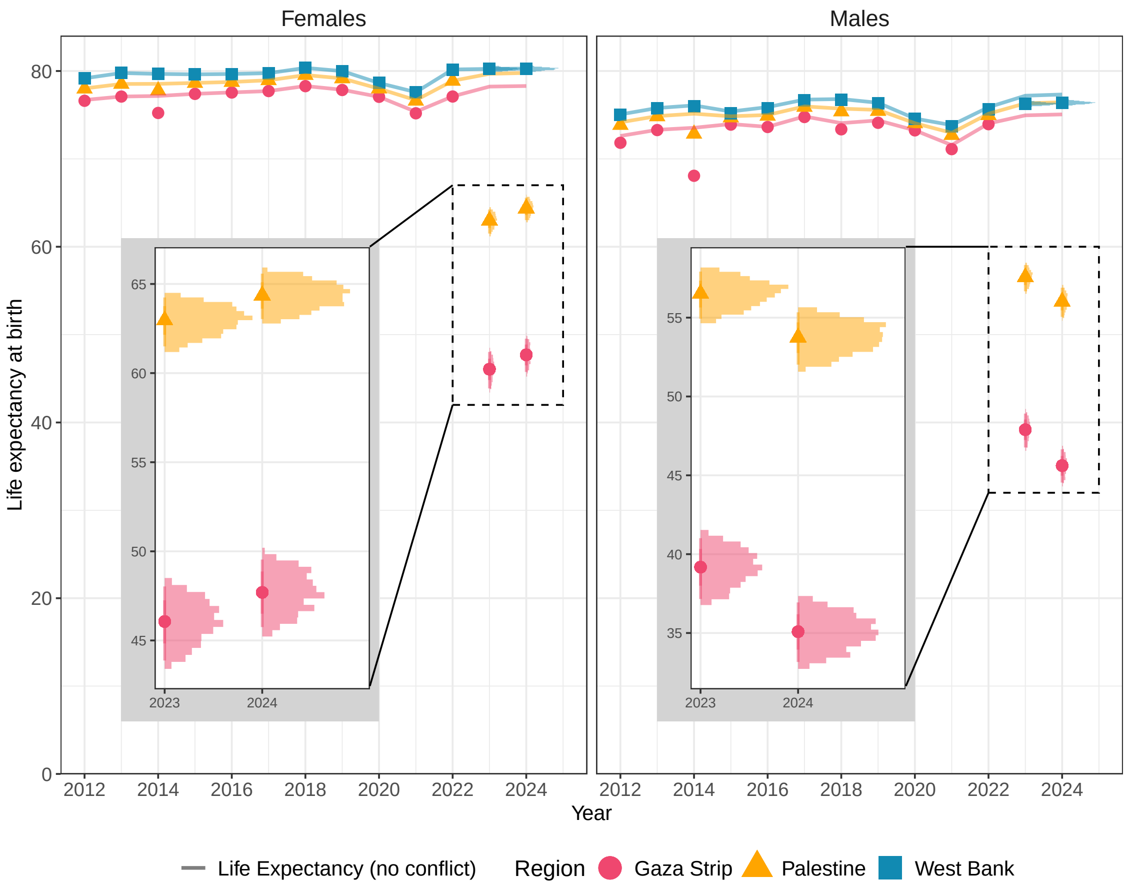


Figure S8: Life expectancy at birth at the national (in yellow) and regional levels using the GMoH age distributions for the Gaza Strip (in pink), and using B'Tselem's age distribution the West Bank (in blue) for 2023 and 2024, with the prior of the reporting rate as defined in the dark blue distribution in Figure S7. The zoomed-in panel shows the results for all of Palestine and the Gaza Strip in 2023-2024. The points indicate the observed life expectancy. The counterfactual scenarios of life expectancy without conflict deaths between 2012 and 2024 are indicated by the lines. For 2023 and 2024, the shapes refer to the mean value, and the histograms summarize the posterior samples of life expectancy estimates.

### 3.3 Sensitivity analysis: reporting rate uncertainty

In this section, we present the results of the sensitivity analysis for the reporting rate. Table S3 contains the estimated LE and loss of LE for the sensitivity analysis described in Section 3.3 of the main text, where we replicated the analysis of Section 3.1 with a different reporting rate prior. The new reporting prior, a shifted and scaled Beta(3,3) distribution, was chosen to reflect the "low" and "high" variants in Guillot et al. (2025).

| Region | Sex | 2012-2019 | 2023 | 2024 |
|---|---|---|---|---|
| **Life expectancy at birth (LE)** | | | | |
| Gaza Strip | Females | 77.2 | 56.3 | 57.5 |
| | | (75.5-78.2) | (53.0-59.0) | (54.4-60.0) |
| | Males | 72.9 | 49.2 | 45.5 |
| | | (68.7-74.6) | (45.8 51.9) | (41.9-48.4) |
| | Total | 75 | 52.5 | 50.7 |
| | | (72-76.2) | (49.1-55.2 | (47.3-53.6) |
| West Bank | Females | 79.7 | 80.2 | 80.3 |
| | | (79.2-80.3) | (79.8-80.6) | (79.9-80.6) |
| | Males | 75.9 | 76.3 | 76.5 |
| | | (75.1-76.7) | (76-76.7) | (76.1-76.8) |
| | Total | 77.8 | 78.2 | 78.3 |
| | | (77.2-78.5) | (78-78.5) | (78-78.6) |
| Palestine | Females | 78.7 | 68.6 | 70.0 |
| | | (77.8-79.5) | (66.7-70.1) | (68.4-71.4) |
| | Males | 74.7 | 62.7 | 62.5 |
| | | (73-75.8) | (60.6-64.4) | (59.1-63.5) |
| | Total | 76.7 | 65.5 | 65.5 |
| | | (75.4-77.4) | (63.5-67.1) | (63.4-67.2) |
| **Life expectancy loss** | | | | |
| Gaza Strip | Females | 0.3 | 21.9 | 20.8 |
| | | (0-1.6) | (19.2-25.2) | (18.3-23.9) |
| | Males | 0.9 | 25.7 | 29.6 |
| | | (0-4.6) | (23.0-29.1) | (26.6-33.2) |
| | Total | 0.6 | 24.2 | 26.0 |
| | | (0-3.2) | (21.5-27.6) | (23.2-29.4) |
| West Bank | Females | 0 | 0 | 0 |
| | | (0-0) | (0-0.4) | (0-0.4) |
| | Males | 0.1 | 0.9 | 0.8 |
| | | (0-0.2) | (0.5-1.2) | (0.5-1.2) |
| | Total | 0.1 | 0.4 | 0.4 |
| | | (0-0.1) | (0.2-0.7) | (0.1-0.7) |
| Palestine | Females | 0.1 | 11.1 | 9.74 |
| | | (0-0.6) | (9.59-13.0) | (8.34-11.4) |
| | Males | 0.4 | 13.6 | 15.0 |
| | | (0-1.9) | (11.9-15.7) | (13.0-17.3) |
| | Total | 0.3 | 12.5 | 12.6 |
| | | (0-1.3) | (10.9-14.5) | (10.9-14.7) |

Table S3: Life expectancy at birth and loss of life expectancy due to conflict in Palestine by sex and region using the sex-age distributions from GMoH for the Gaza Strip and B'Tselem for the West Bank, with the prior of the reporting rate based on a shifted an scaled beta distribution (Beta(3,3)*0.48 + 0.76; see Figure 3 in the main text), which is centered at 1.0 and allows for underreporting and overreporting. Life expectancy loss is estimated from a counterfactual based on Lee-Carter projections of mortality in the absence of the conflict. The intervals represent the 95% credible intervals.

## 3.4 Comparison to other studies

In this section, we describe additional analyses performed on the cumulative time period between Oct. 7th 2023 and Oct. 6th 2024, following the analysis described in Guillot et al. (2025). To investigate how much of the uncertainty in the life expectancy estimates is due to the reporting rate, we set up the following reporting rate prior using the "variant" scenarios in Guillot et al. (2025). The light blue distribution in Figure S7 shows the Beta distribution we selected based on these variants, where the bounds of the distributed are set at 76% underreporting and 124% overreporting.

Figure S9 shows the estimated life expectancies and loss of life expectancies for the total cumulative period from Oct. 2023 - Dec. 2024. We again see clear differences in estimated life expectancies for females between the B'Tselem historical averages and the UN-IGME genocide/GMoH age distributions, while male life expectancies converge more closely. For total life expectancy and loss of life expectancy, the estimates essentially overlap, which follows the results in the main text.

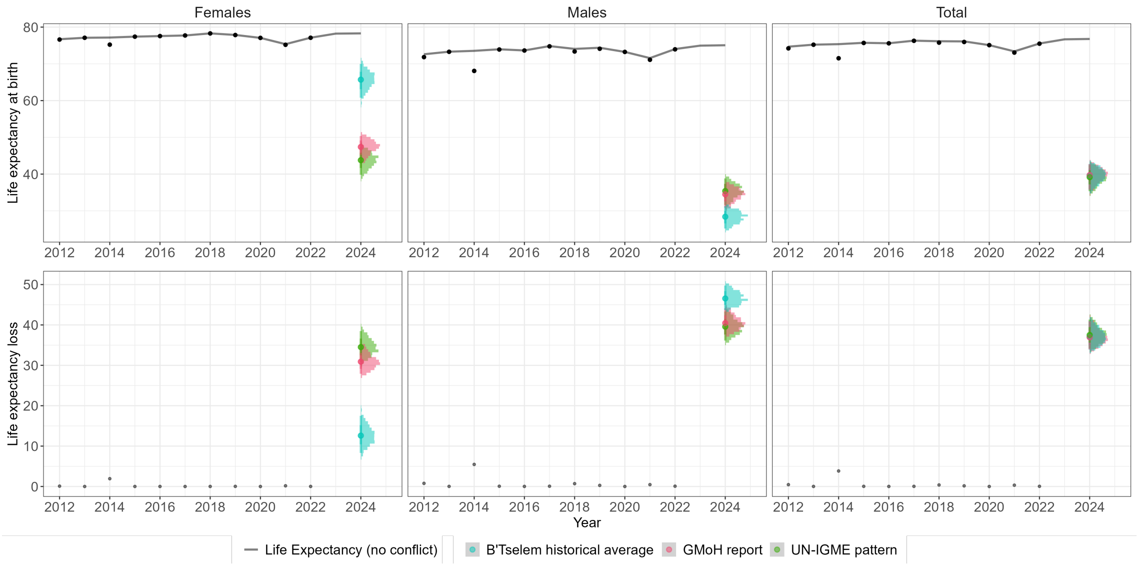


Figure S9: Life expectancy at birth for the Gaza Strip in for the one year Oct 2023- Oct 2024 period using the age distributions from 1) the GMoH (in pink) 2) B'Tselem historical average (in blue) 3) UN-IGME genocide crisis pattern (in green), with the prior of the reporting rate following the different variant scenarios described in Guillot et al. (2025). The points refer to the mean value and the histograms summarize the posterior samples of life expectancy estimates.

### 3.5 Extended results using the GMoH distribution

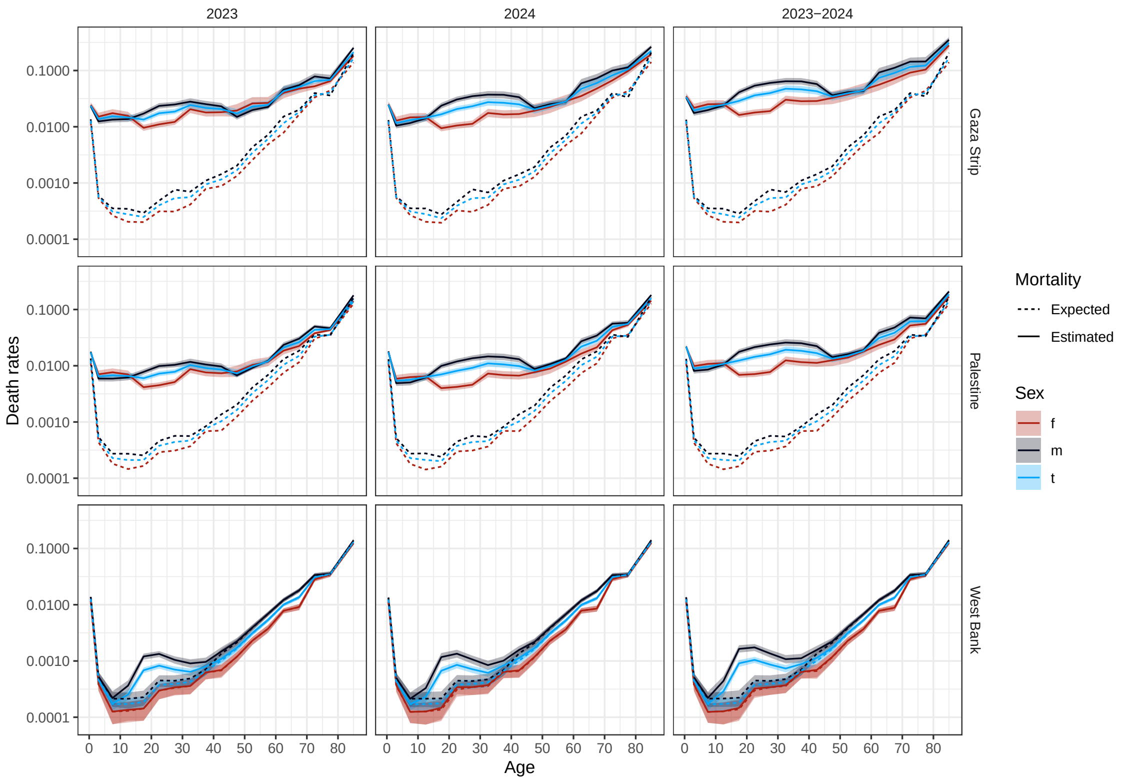


Figure S10: Death rates by region, age and sex using the age distributions from the GMoH. The dashed line refers to the baseline mortality (estimated mortality in the absence of conflict) and the solid line to the estimated mortality, including conflict and non-conflict. The 2023-2024 results refer to the period October 7, 2023-October 6, 2024.

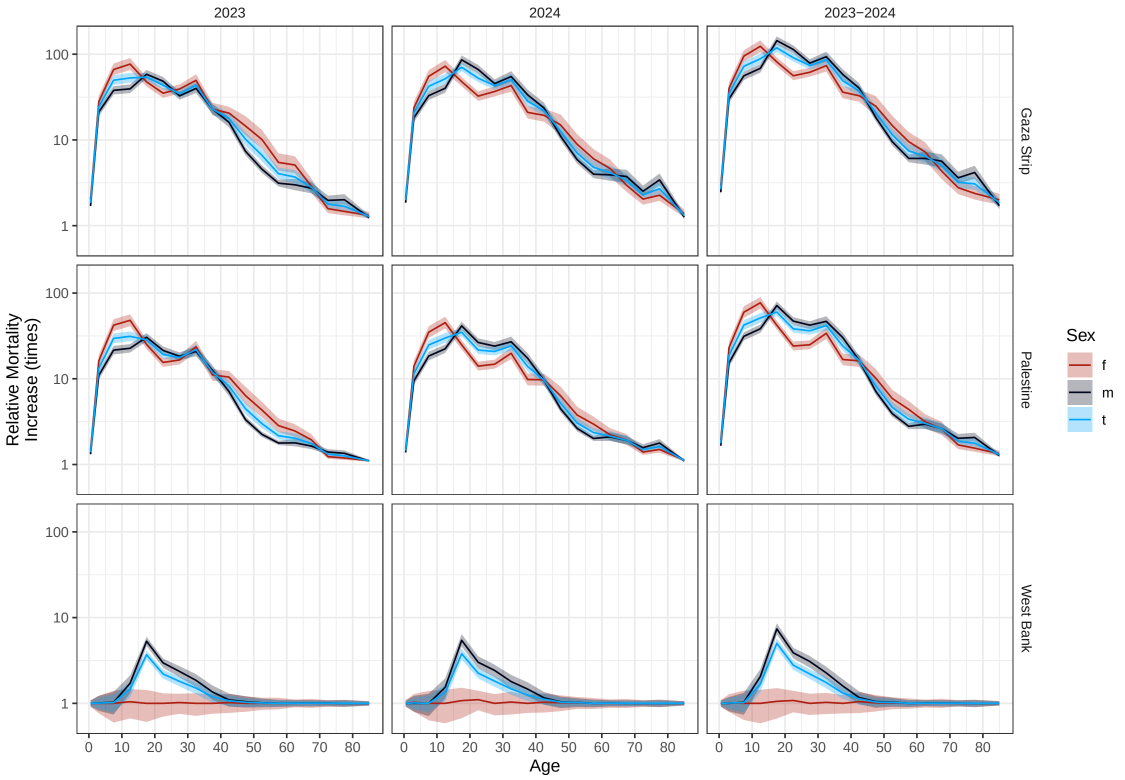


Figure S11: Relative mortality increase by region, age and sex using the age distributions from the GMoH. The relative mortality is calculated by dividing the estimated mortality, including conflict and non-conflict, by the baseline mortality (estimated mortality in the absence of conflict). The 2023-2024 results refer to the period October 7, 2023-October 6, 2024.

| Region | Year | Sex | Total deaths | Conflict-related deaths | Relative mortality increase (times) |
|---|---|---|---|---|---|
| Gaza Strip | 2023 | Females | 20329<br>(17729, 23657) | 17552<br>(14953, 20880) | 7.60<br>(6.60, 8.80) |
| | | Males | 24178<br>(21960, 26769) | 20025<br>(17806, 22616) | 7.40<br>(6.70, 8.20) |
| | | Total | 44507<br>(39737, 50408) | 37577<br>(328076, 43478) | 7.50<br>(6.70, 8.50) |
| | 2024 | Females | 18237<br>(15840, 21380) | 15739<br>(13342, 18882) | 7.30<br>(6.30, 8.60) |
| | | Males | 28002<br>(25171, 31453) | 25002<br>(22172, 28454) | 9.30<br>(8.40, 10.50) |
| | | Total | 46239<br>(41049, 52858) | 40741<br>(35551, 47360) | 8.40<br>(7.50, 9.60) |
| | 2023-2024 | Females | 30498<br>(26278, 36000) | 27811<br>(23592, 33313) | 11.80<br>(10.20, 13.90) |
| | | Males | 48186<br>(43232, 541540) | 44160<br>(39206, 50128) | 15.40<br>(13.80, 17.30) |
| | | Total | 78684<br>(69499, 90143) | 71971<br>(62786, 83430) | 13.80<br>(12.20, 15.80) |
| Palestine | 2023 | Females | 24343<br>(21692, 27615) | 17690<br>(15038, 20962) | 3.70<br>(3.30, 4.20) |
| | | Males | 29413<br>(27119, 32067) | 20482<br>(18188, 23137) | 3.70<br>(3.40, 4.00) |
| | | Total | 53756<br>(48815, 59648) | 38172<br>(33231, 44064) | 3.70<br>(3.30, 4.10) |
| | 2024 | Females | 22548<br>(20139, 25612) | 16079<br>(13669, 19143) | 3.50<br>(3.10, 4.00) |
| | | Males | 33463<br>(30599, 36852) | 25539<br>(22675, 28928) | 4.20<br>(3.90, 4.70) |
| | | Total | 56011<br>(50755, 62426) | 41617<br>(36361, 48032) | 3.90<br>(3.50, 4.30) |
| | 2023-2024 | Females | 34712<br>(30326, 40108) | 28101<br>(23715, 33497) | 5.30<br>(4.70, 6.20) |
| | | Males | 53863<br>(48681, 59860) | 44987<br>(39804, 50983) | 6.80<br>(6.10, 7.50) |
| | | Total | 88575<br>(79067, 99939) | 73088<br>(63580, 84452) | 6.10<br>(5.50, 6.90) |
| West Bank | 2023 | Females | 4040<br>(3910, 4173) | 2<br>(0, 6) | 1.00<br>(1.00, 1.00) |
| | | Males | 5385<br>(5236, 5520) | 341<br>(307, 380) | 1.10<br>(1.10, 1.10) |
| | | Total | 9425<br>(9219, 9613) | 344<br>(309, 383) | 1.10<br>(1.00, 1.10) |
| | 2024 | Females | 4144<br>(4014, 4263) | 15<br>(7, 23) | 1.00<br>(1.00, 1.00) |
| | | Males | 5510<br>(5354, 5660) | 540<br>(488, 603) | 1.10<br>(1.10, 1.10) |
| | | Total | 9654<br>(9467, 9844) | 555<br>(501, 620) | 1.10<br>(1.00, 1.10) |
| | 2023-2024 | Females | 4099<br>(3979, 4220) | 14<br>(6, 22) | 1.00<br>(1.00, 1.00) |
| | | Males | 5703<br>(5535, 5857) | 788<br>(710, 875) | 1.20<br>(1.10, 1.20) |
| | | Total | 9802<br>(9609, 9999) | 802<br>(723, 892) | 1.10<br>(1.10, 1.10) |

Table S4: Total estimated deaths, conflict-related deaths and relative mortality increase by region, sex and time period. The total deaths columns includes conflict and non-conflict deaths. The relative mortality is calculated by dividing the estimated mortality, including conflict and non-conflict, by the baseline mortality (estimated mortality in the absence of conflict). The 2023-2024 results refer to the period Oct 2023-Oct 2024.

### 3.6 Including COVID-19 period

In this section, we present sensitivity analyses from forecasting the (pre-war) non-conflict mortality in Gaza including the COVID period (2012-2022), using the same age-sex distributions shown in Figure S6. Figure S12 shows the estimated life expectancies and loss of life expectancies. Compared to Figure S6, estimated mean life expectancy for females using the 2012-2022 fitting period is slightly higher using the historical (B'tselem) age distribution, with an estimated mean of 67.5 years compared to the previous estimate of 65.7 years. The estimated male life expectancy in Figure S12 was also slightly lower than the previous estimate in Figure S6, with an estimated mean of 30.5 compared to the previous mean estimate of 31.5. The differences between all other scenarios including the 2020-2022 period were all within 1 year.

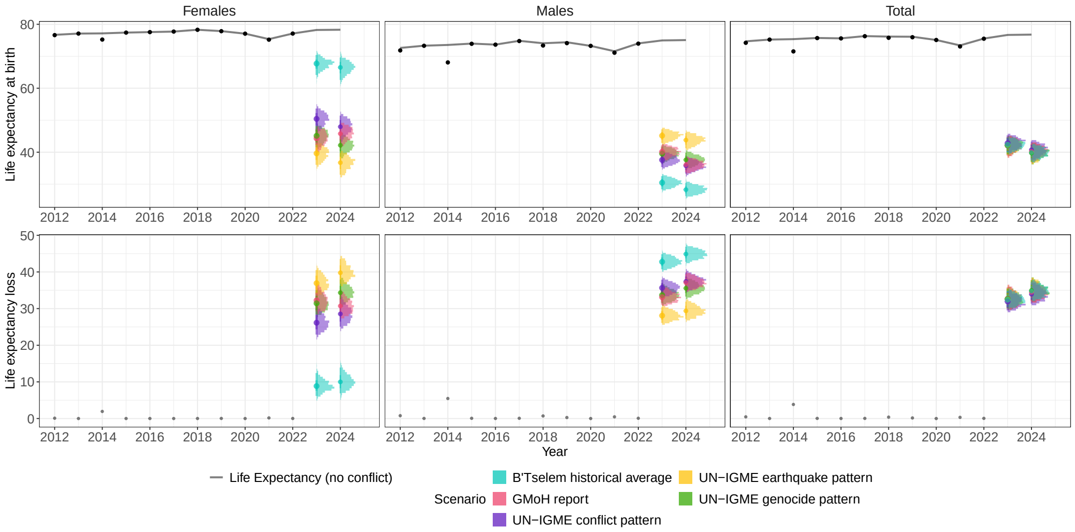


Figure S12: Life expectancy at birth and loss of life expectancy estimates for Gaza using the age distributions from 1) the GMoH 2) B'Tselem historical average 3) UN-IGME genocide crisis pattern 4) UN-IGME conflict crisis pattern and 5) UN-IGME earthquake crisis pattern, using forecasted non-conflict mortality up to 2022 (including COVID-19).

## References


Airwars (2024). Methodology note: Civilian harm from explosive weapons use in Gaza. `https://docs.google.com/spreadsheets/d/1DpyvHQlMAug43wY1ydbeIvIF01rcH4_-aMT9p-AWUVQ/edit?gid=109569791#gid=109569791`. Accessed: March 1, 2025.

Gaza Ministry of Health (GMoH) (2023). Detailed report of the victims of israel's attack on gaza strip from october 7th to october 26th, 2023. `https://www.palestinechronicle.com/wp-content/uploads/2023/10/here.pdf`.

Gelman, A., Bois, F., and Jiang, J. (1996). Physiological pharmacokinetic analysis using population modeling and informative prior distributions. *Journal of the American Statistical Association*, 91(436):1400–1412.

Guillot, M., Draidi, M., Cetorelli, V., Silva, J. H. C. M. D., and Lubbad, I. (2025). Life expectancy losses in the Gaza Strip during the period October, 2023, to September, 2024. *The Lancet*, 405(10477):478–485. Publisher: Elsevier.

Israeli Information Center for Human Rights in the Occupied Territories (B'Tselem) (2023). Database on fatalities and house demolitions. `https://statistics.btselem.org/en`.

Jamaluddine, Z., Abukmail, H., Aly, S., Campbell, O. M. R., and Checchi, F. (2025). Traumatic injury mortality in the Gaza Strip from Oct 7, 2023, to June 30, 2024: a capture–recapture analysis. *The Lancet*, 405(10477):469–477. Publisher: Elsevier.

Jamaluddine, Z., Chen, Z., Abukmail, H., Aly, S., Elnakib, S., Barnsley, G., and et. al (2024). Crisis in Gaza: Scenario-based health impact projections.

Mathers, C., Castanheira, H. C., Sohn, H., You, D., Hug, L., Pelletier, F., and Gerland, P. (2023). Age-Sex Patterns of Crisis Deaths: Towards a more standard mortality estimation approach. *Working Paper, United Nations Children's Fund, New York.*

Tech for Palestine (2025). Daily Casualties- Gaza. https://data.techforpalestine.org/docs/casualties-daily/.

United Nations Office for the Coordination of Humanitarian Affairs (OCHA) (2023). Hostilities in the Gaza Strip and Israel - reported impact: Day 85. http://www.ochaopt.org/content/hostilities-gaza-strip-and-israel-reported-impact-day-85.

United Nations Office for the Coordination of Humanitarian Affairs (OCHA) (2024a). Data on casualties. https://www.ochaopt.org/data/casualties.

United Nations Office for the Coordination of Humanitarian Affairs (OCHA) (2024b). *Reported impact snapshot: Gaza Strip (31 December 2024).*
